# Supplementary material for: Structural and dynamic mechanisms for coupled folding and tRNA recognition of a translational T-box riboswitch
Source: Nat Commun. 2023 Nov 15;14:7394. doi: 10.1038/s41467-023-43232-z (PMC10651847; doi:10.1038/s41467-023-43232-z)
Supplement: Supplementary file 1 — Supplementary Information [file 41467_2023_43232_MOESM1_ESM.pdf]

Supplementary Information

Structural and dynamic mechanisms for coupled folding and tRNA recognition in a translational T-box riboswitch

Xiaolin Niu[1], Zhonghe Xu[1], Yufan Zhang[2], Xiaobing Zuo[3], Chunlai Chen[1, 4, *], Xianyang Fang[1, 2, *]

[1]Beijing Frontier Research Center for Biological Structure, School of Life Sciences, Tsinghua University, Beijing 100084, China.

[2]Key Laboratory of RNA Science and Engineering, Institute of Biophysics, Chinese Academy of Sciences, Beijing, 100101, China

[3]X-ray Science Division, Argonne National Laboratory, Lemont IL 60439, USA.

[4]State Key Laboratory of Membrane Biology, Tsinghua University, Beijing 100084, China.

[*]e-mail: chunlai@mail.tsinghua.edu.cn; fangxy@ibp.ac.cn

Table of Contents

Supplementary Methods 3

Supplementary Figure 1 28

Supplementary Figure 2 29

Supplementary Figure 3 30

Supplementary Figure 4 31

Supplementary Figure 5 32

Supplementary Figure 6 33

Supplementary Figure 7 34

Supplementary Figure 8 35

Supplementary Figure 9 36

Supplementary Table 1 37

Supplementary Table 2 37

Supplementary Table 3 38

Supplementary Table 4 39

Supplementary Table 5 40

Supplementary Table 6 41

Supplementary Table 7 42

Supplementary References..43

Supplementary Methods

Synthetic procedures and characterizations of rNaM^{CO}TP

General

All solvents and reagents were purchased commercially and used without further purification. For synthetic procedures, all reactions were carried out in oven-dried glassware under an inert atmosphere. Solvents were distilled and/or dried over 4 Å molecular sieves. NMR spectra were recorded on an AVANCE III 1 BAY 400 MHz Bruker NMR spectrometer.

Synthetic schemes and procedures

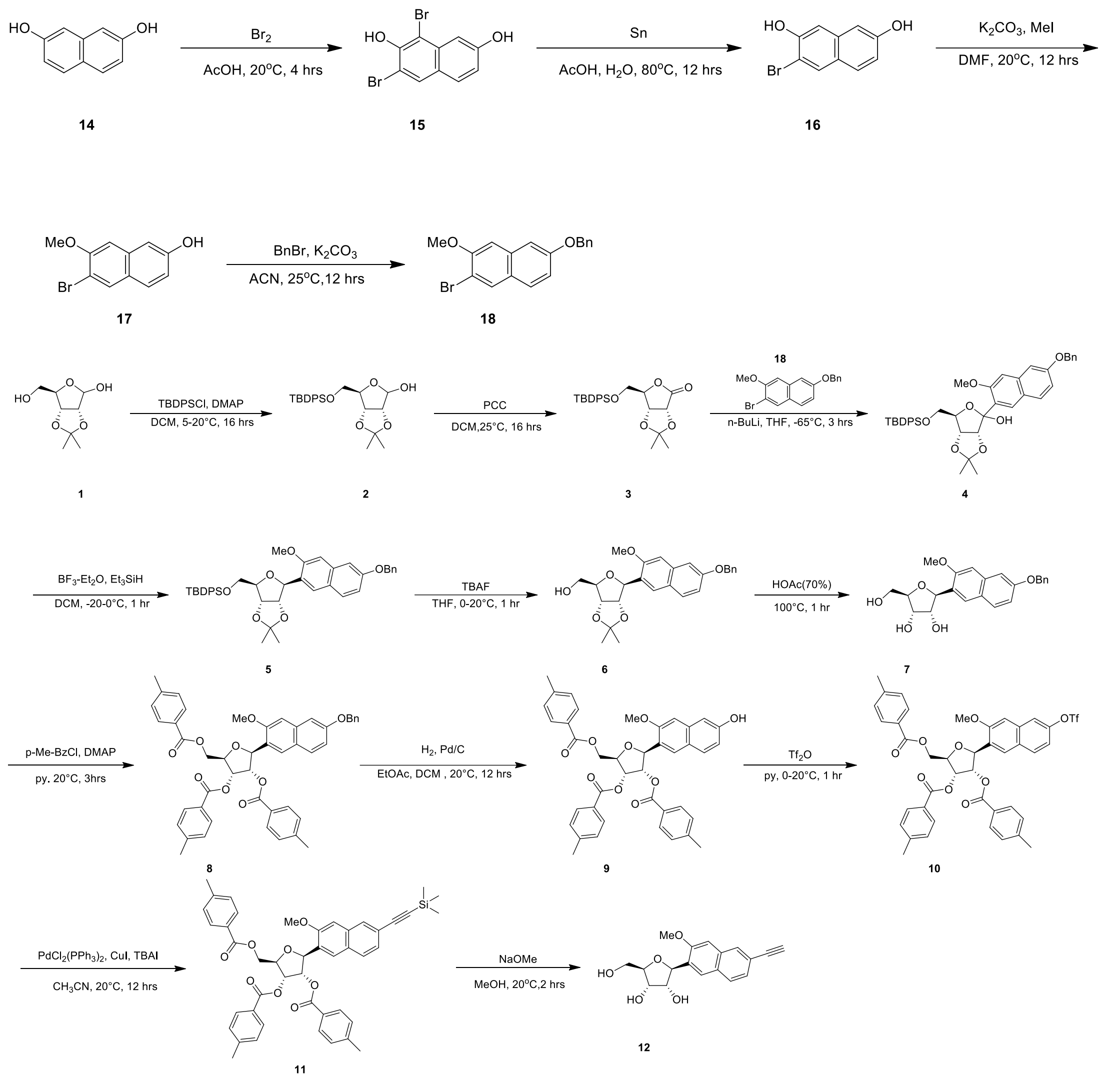

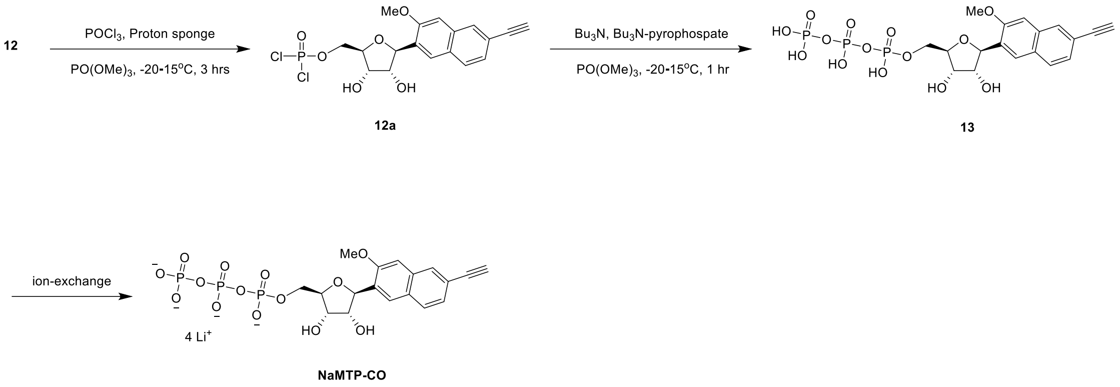

General procedure for preparation of compound 15

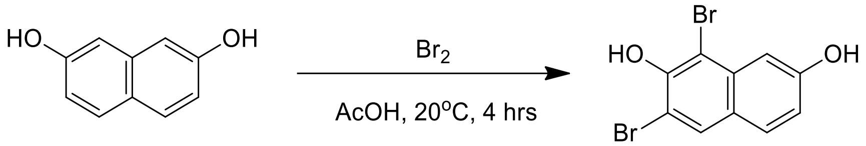

14 **15**

To a solution of compound **14** (100 g, 624 mmol, 1.00 *eq*) in AcOH (600 mL) was added Br_2 (199 g, 1.25 mol, 64.3 mL, 2.00 *eq*) in AcOH (400 mL). The mixture was stirred at 20 °C for 4 hrs. TLC (petroleum ether/ethyl acetate = 2/1, R_f = 0.50) indicated compound **14** was consumed completely and one new spot formed. Compound **15** (595 g, 1.87 mol, 99.9% yield)（reaction solution）was obtained as a brown liquid.

TLC: petroleum ether/ethyl acetate = 2/1, R_f = 0.50

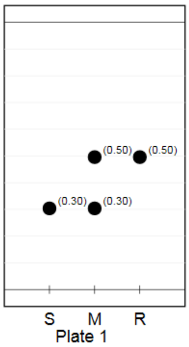

General procedure for preparation of compound 16

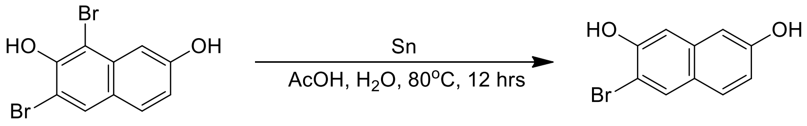

15 **16**

To a solution of compound **15** (198 g, 624 mmol, 1.00 *eq*) in H_2O (400 mL) was added Sn (154 g, 1.30 mol, 21.0 mL, 2.08 *eq*). The mixture was stirred at 80 °C for 12 hrs. TLC (petroleum ether/ethyl acetate = 2/1, R_f = 0.34) indicated compound **15** was

consumed completely and one new spot formed. Combine three batches. The reaction mixture was concentrated under reduced pressure to give a residue. The crude product was triturated with H_2O (2 L) at 25 °C for 1 hr, the mixture was filtered and get filter cake. Filter cake was triturated with toluene (2 L) at 25 °C for 1 hr, the mixture was filtered and get filter cake. Concentrated under reduced pressure to give a residue. Compound **16** (336 g, 1.41 mol, 75.0% yield) was obtained as a white solid.

TLC: petroleum ether/ethyl acetate = 2/1, $R_f = 0.34$

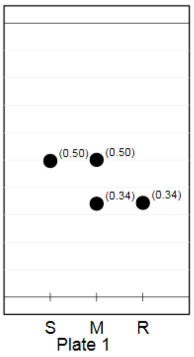

^{1}H NMR: (ET38781-33-P1A1, 400 MHz, DMSO)

δ ppm 10.37 (s, 1H), 9.71 (s, 1H), 7.97 (s, 1H), 7.60 (d, J = 8.8 Hz, 1H), 7.08(s, 1H), 6.90-6.83(m, 2H)

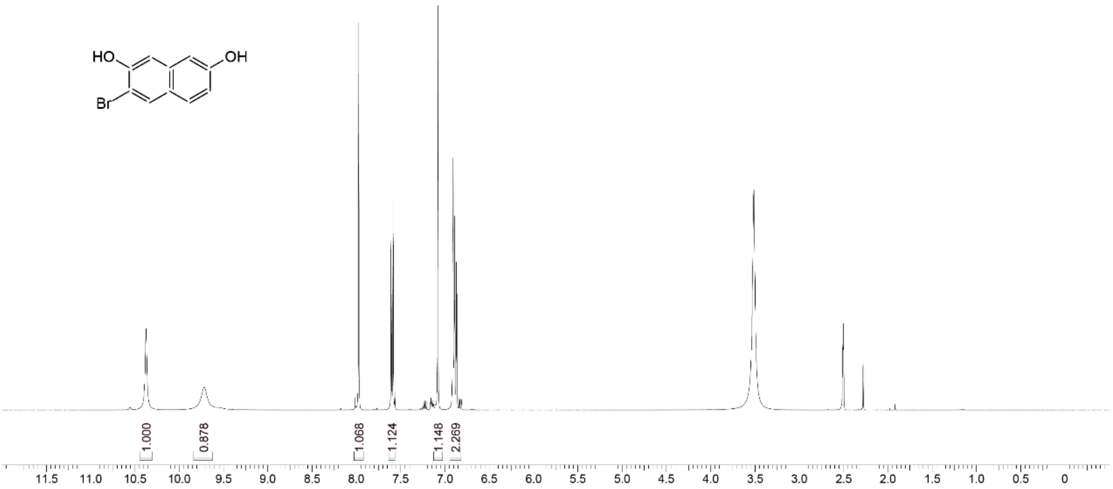

General procedure for preparation of compound 17

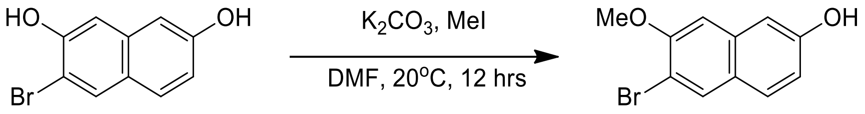

16 17

Compound **16** (168 g, 702 mmol, 1.00 *eq*) was added to a suspension of K_2CO_3 (194 g, 1.41 mol, 2.00 *eq*) in DMF (1680 mL) and the mixture was stirred at 20 °C under N_2.

Added MeI (119 g, 843 mmol, 52.5 mL, 1.20 *eq*) and the mixture was stirred at 20 °C for 12 hrs, TLC (petroleum ether/ethyl acetate = 2/1, $R_f = 0.60$) indicated compound **16** was consumed completely and one main new spot formed. Combine with two batches. The reaction mixture was filtered through celite and concentrated under reduced pressure to give a residue. The residue was purified by column chromatography (SiO_2, petroleum ether/ethyl acetate = 50/1 to 1/1). Compound **17** (183.6 g, 725 mmol, 51.6% yield) was obtained as a white solid.

TLC: petroleum ether/ethyl acetate = 2/1, $R_f = 0.60$

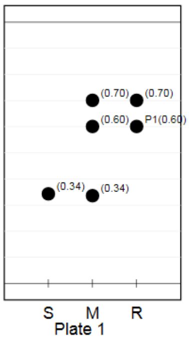

^{1}H NMR: (ET38781-36-P1A1, 400 MHz, MeOD)

δ ppm 7.89 (s, 1H), 7.54 (d, J = 8.8 Hz, 1H), 7.08 (s, 1H), 7.04 (d, J = 2.4 Hz, 1H), 6.92 (dd, J = 2.4 Hz, 9.6 Hz, 1H), 3.92 (s, 3H).

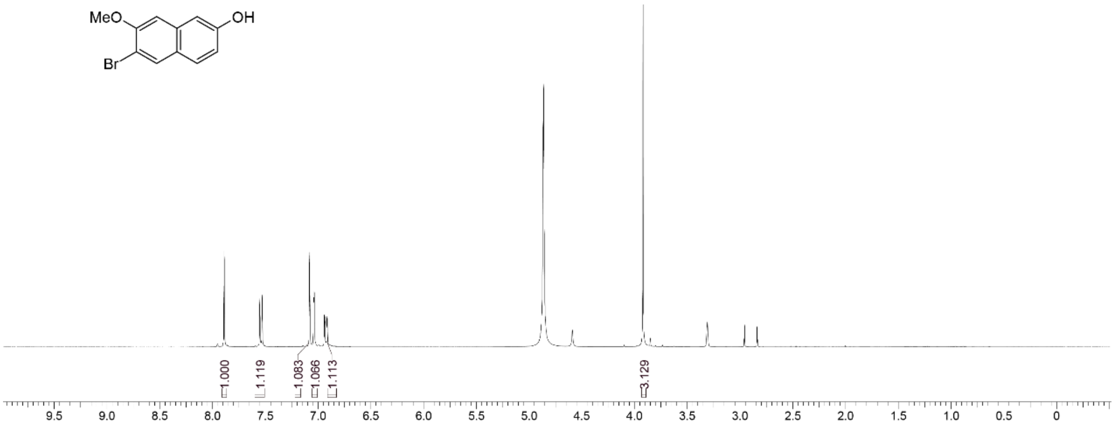

General procedure for preparation of compound 18

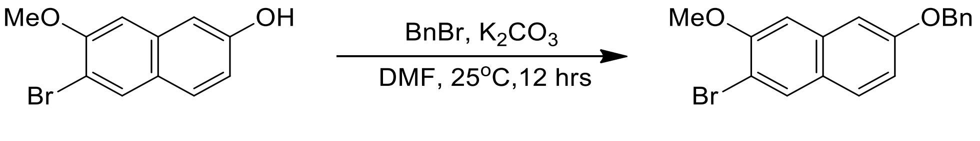

17 **18**

To a solution of compound **17** (91.8 g, 362.71 mmol, 1.00 *eq*) in DMF (550 mL) was added K_2CO_3 (75.1 g, 544 mmol, 1.50 *eq*) and BnBr (68.2 g, 398 mmol, 47.3 mL, 1.10

eq). The mixture was stirred at 25 °C for 12 hrs. TLC (petroleum ether/ethyl acetate = 5/1, R_f = 0.84) indicated compound **17** was consumed completely and two new spots formed. Combine two batches. The reaction mixture was filtered through Celite and concentrated under reduced pressure to give a residue. The residue was purified by prep-HPLC (neutral condition; column: Agela DuraShell C18 250 x 80 mm x 10 um; mobile phase: [water (10 mM NH_4HCO_3)-ACN]; B%: 0%-10%, 20 min). Compound **18** (160 g, 466.18 mmol, 64.2% yield) was obtained as a white solid.

TLC: petroleum ether/ethyl acetate = 5/1, R_f = 0.84

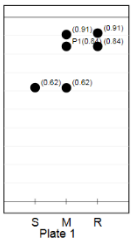

^{1}H NMR: (ET38781-38-P1A1, 400 MHz, $CDCl_3$)

δ ppm 7.87 (s, 1H), 7.51 (d, *J* = 8.4 Hz, 1H), 7.39 (d, *J* = 7.2 Hz, 2H), 7.34-7.30 (m, 2H), 7.28-7.24 (m, 1H),7.02 (s, 1H), 7.00 (d, *J* =2.4Hz, 1H), 6.95 (s, 1H), 5.07 (s, 2H), 3.88 (s, 3H).

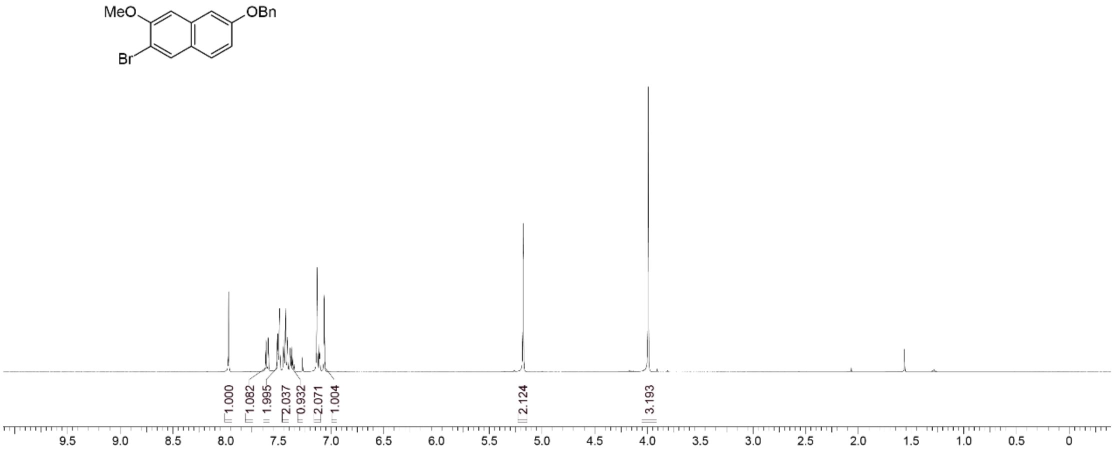

General procedure for preparation of compound 2

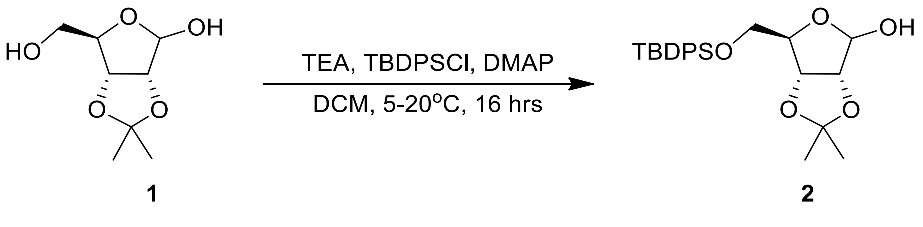

To a solution of compound **1** (200 g, 1.05 mol, 1.00 *eq*) in DCM (1200 mL) was add TEA (212 g, 2.10 mol, 292 mL, 2.00 *eq*), and DMAP (6.42 g, 52.5 mmol, 0.05 *eq*), TBDPSCl (317g, 1.16 mol, 297 mL, 1.10 *eq*) dropwised at 5-10 °C, then the mixture

was stirred at 20 °C for 16 hrs. TLC (petroleum ether/ethyl acetate = 3/1, R_f = 0.6) indicated of compound **1** was not remained, and one major new spot was detected. Combine two batches. The mixture was poured to 2000 mL ice-water, stirred for 10 mins, separated organic layer was washed with 700 mL brine, dried with Na_2SO_4, filtered and concentrated to give crude product. The residue was purified by column chromatography (SiO_2, petroleum ether/ethyl acetate = 100/1 to 10/1). Compound **2** (510 g, 1.19 mol, 56.5% yield) was obtained as a yellow oil.

TLC: petroleum ether/ethyl acetate 3/1, R_f = 0.6

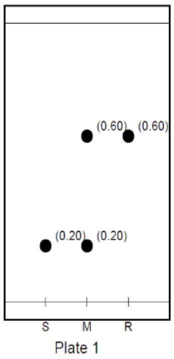

^{1}H NMR: ET38760-5-P1A1, 400 MHz, $CDCl_3$

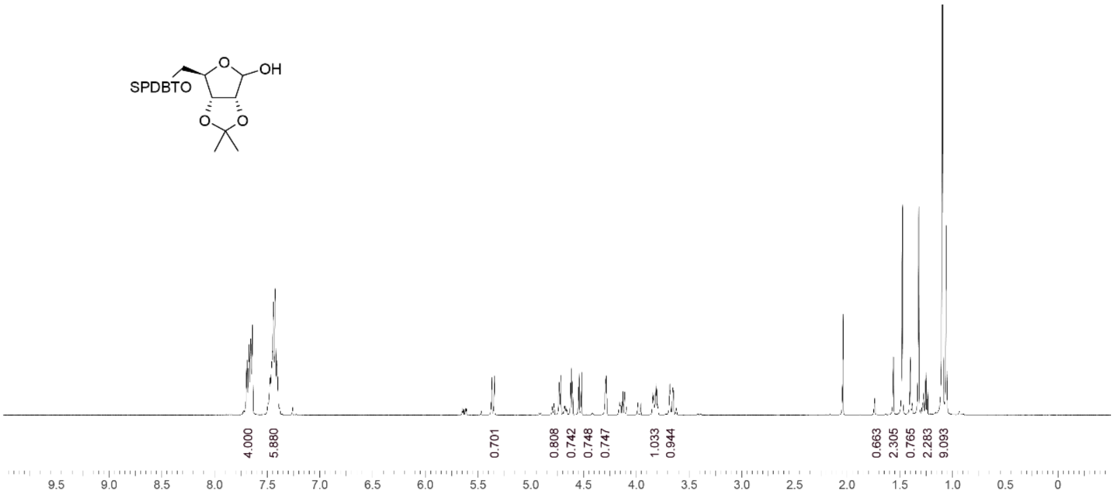

General procedure for preparation of compound 3

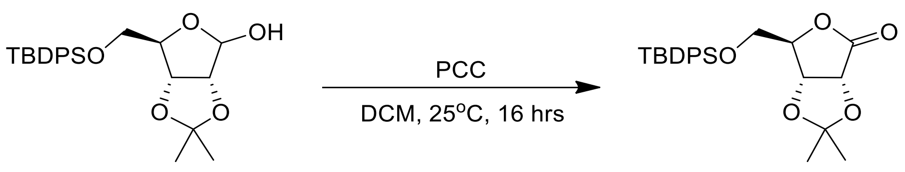

2 **3**

To a solution of compound **2** (135 g, 314 mmol, 1.00 *eq*) in DCM (810 mL) was added PCC (101 g, 472 mmol, 1.50 *eq*) with celatom stirred, the mixture was stirred at 25 °C

for 16 hrs. TLC (petroleum ether/ethyl acetate = 5/1, R_f = 0.64) indicated compound **2** was not remained, and one major new spot was detected. LCMS (ET38911-13-P1A1, RT = 1.009 min) showed compound **2** was consumed completely and one main peak with desired m/z. Combine two batches. Filter and add H_2O (3000 mL) to the mixture, extract the mixture with DCM (2000 mL), separate the mixture. Wash the organic layer with brine (1200 mL), separate the mixture. Dry over Na_2SO_4, filter. The reaction mixture was concentrated under reduced pressure. The residue was purified by column chromatography (SiO_2, petroleum ether/ethyl acetate=50/1 to 10/1)**.** Compound **3** (215 g, 504 mmol, 80.0% yield) was obtained as a white solid.

TLC: petroleum ether/ethyl acetate = 5/1, R_f = 0.64

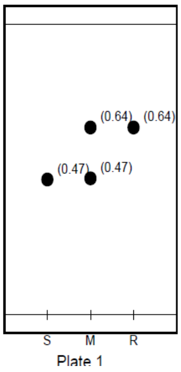

^{1}H NMR: ET38911-13-P1B1, 400 MHz, DMSO

δ ppm 7.65-7.50 (m, 2H), 7.49-7.48 (m, 2H), 7.48-7.38 (m, 6H), 4.94 (d, *J* = 5.6Hz, 1H), 4.80 (d, *J* = 5.6Hz, 1H), 4.72 (s, 1H), 3.99 (d, *J* = 11.6Hz, 1H), 3.78 (dd, *J* = 11.6Hz, *J* = 2.4Hz, 1H), 1.38 (s, 3H), 1.35 (s, 3H), 0.97 s, 9H)

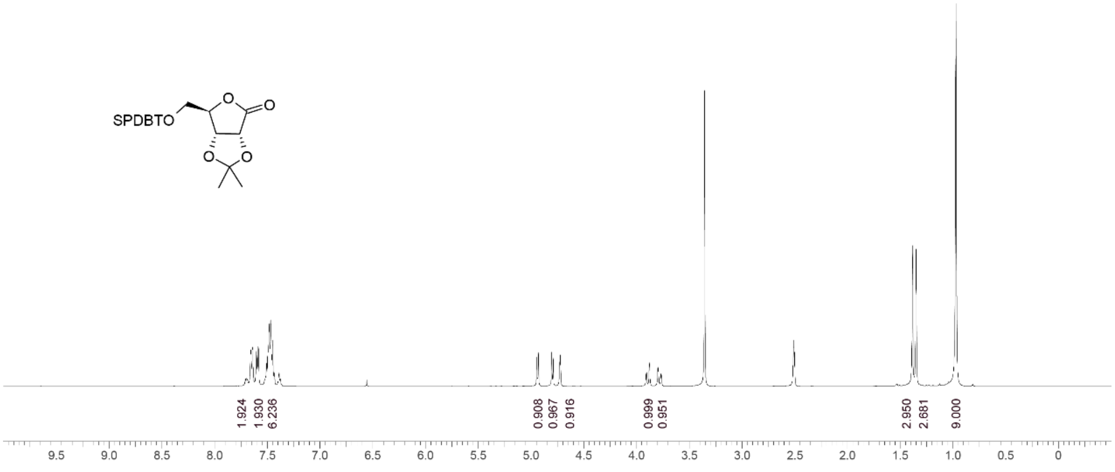

General procedure for preparation of compound 4

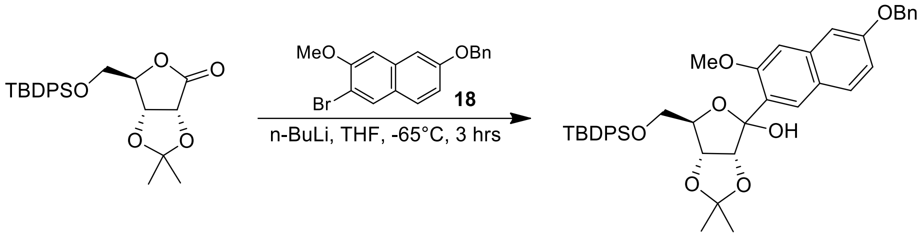

3 4

To a solution of compound **18** (70.0 g, 203 mmol, 1.00 *eq*) in THF (490 mL) was added n-BuLi (2.5 M, 97.90 mL, 1.20 *eq*) dropwise at -65 °C under N_2. The mixture was stirred at -65 °C for 1 hr. Then, add compound **3** (87.0 g, 203 mmol, 1.00 *eq*) in THF (300 mL) dropwise to the mixture at -65 °C. The mixture was stirred at -65 °C for 2 hrs. TLC (petroleum ether/ethyl acetate = 10/1, R_f = 0.05) showed the reaction was complete. The mixture was poured into saturate aq. NH_4Cl (800 mL), extracted with ethyl acetate (800 mL x 2), concentrated in vacuum to give residue. The residue was purified by column chromatography (SiO_2, petroleum ether/ethyl acetate=100/1 to 10/1). Compound **4** (84.2 g, 121 mmol, 59.7% yield) was obtained as a yellow oil.

TLC: petroleum ether/ethyl acetate = 10/1, R_f = 0.05

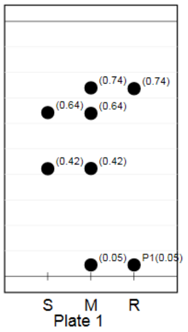

^{1}H NMR: ET38781-37-P1A1, 400 MHz, $CDCl_3$

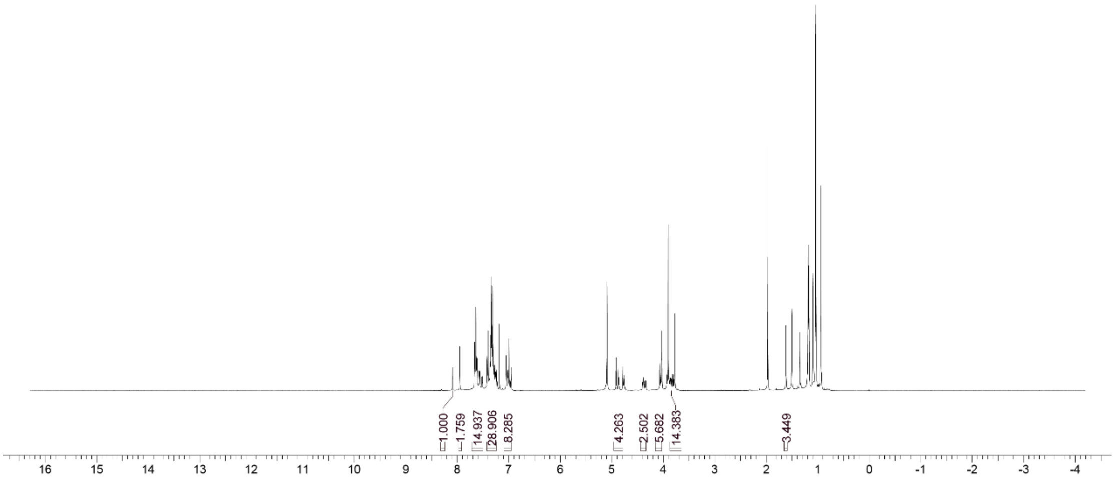

General procedure for preparation of compound 5

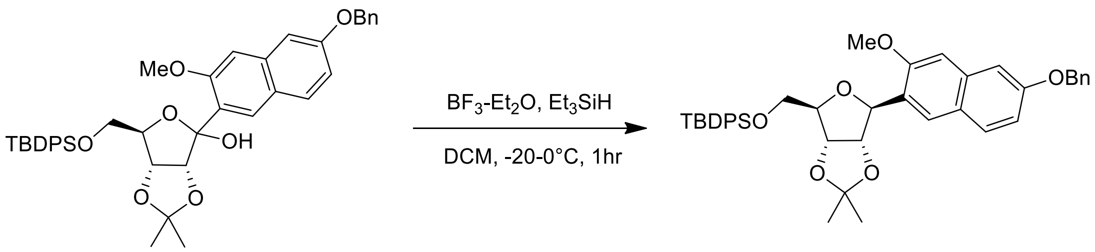

4 **5**

To a solution of compound **4** (55.6 g, 80.5 mmol, 1.00 *eq*) in DCM (340 mL) was added Et_3SiH (18.7 g, 161 mmol, 25.7 mL, 2.00 *eq*) and $BF_3.Et_2O$ (13.7 g, 96.6 mmol, 11.9 mL, 1.20 *eq*). The mixture was stirred at -20-0 °C for 1 hr. TLC (petroleum ether/ethyl acetate = 5/1, R_f = 0.50) indicated compound **4** was consumed completely and a new spot formed. Combine two batches. The mixture was poured into saturate aq. $NaHCO_3$ (600 mL), extracted with DCM (600 mL x 2), concentrated in vacuum. The residue was purified by column chromatography (SiO_2, Petroleum ether/Ethyl acetate=1/0 to 10/1). Compound **5** (63.0 g, 93.3 mmol, 57.9% yield) was obtained as a yellow oil.

TLC: petroleum ether/ethyl acetate = 5/1, R_f = 0.50

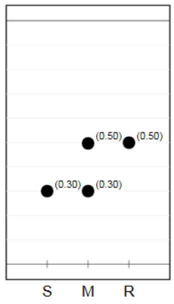

General procedure for preparation of compound 6

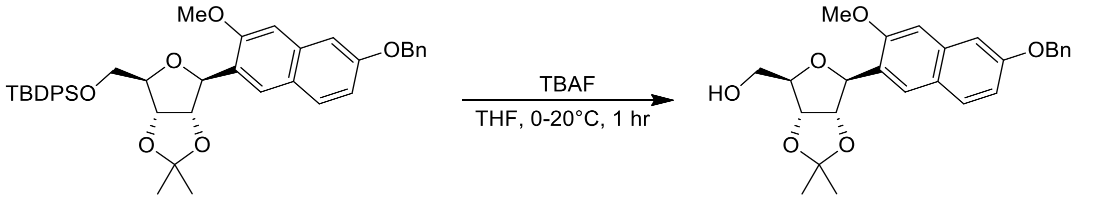

5 **6**

To a solution of compound **5** (56.0 g, 82.9 mmol, 1.00 *eq*) in THF (340 mL) was added TBAF (1.0 M, 99.5 mL, 1.20 *eq*) at 0 °C. The mixture was stirred at 20 °C for 1 hr. TLC (petroleum ether/ethyl acetate = 3/1, R_f = 0.19) indicated compound **5** was consumed completely and two new spots formed. The mixture was poured into water (400 mL), extracted the mixture with ethyl acetate (400 mL x 2), concentrated in vacuum. The residue was purified by column chromatography (SiO_2, petroleum ether/ethyl acetate = 1/0 to 10/1**)**. Compound **6** (14.0 g, 32.0 mmol, 38.6% yield) was

obtained as a colorless oil

TLC: petroleum ether/ethyl acetate = 3/1, $R_f = 0.19$

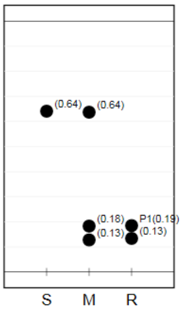

^{1}H NMR: ET38781-41-P1A1, 400 MHz, $CDCl_3$

δ ppm 7.70-7.66 (m, 2H), 7.50-7.48 (m, 2H), 7.42-7.40 (m, 3H), 7.15-7.08 (m, 3H), 5.18 (s, 2H), 5.13-4.94 (m,1H), 4.93-4.89 (m, 2H), 4.22-4.21 (m, 1H), 4.14-4.12 (m, 4H), 3.96-3.81 (m, 1H), 2.59(d, J = 7.6 Hz， 1H), 1.67 (s, 3H), 1.37 (s, 3H)

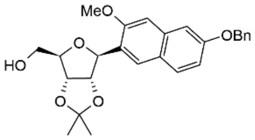

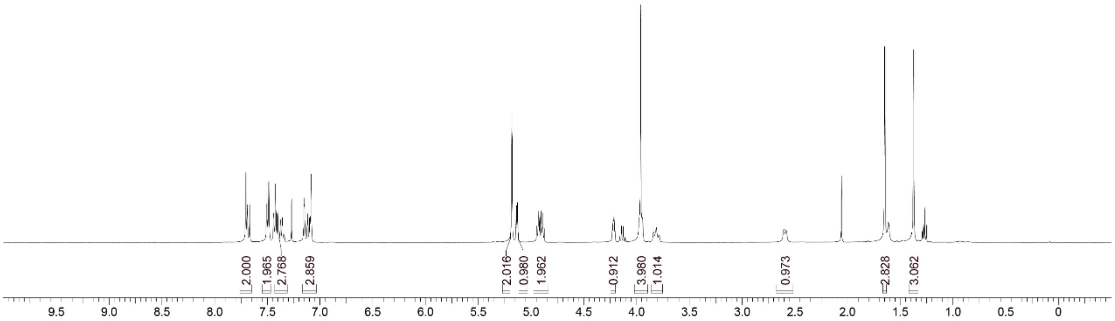

General procedure for preparation of compound 7

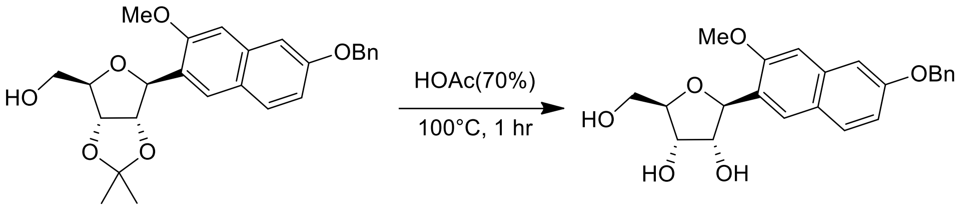

6 7

To a solution of compound **6** (15.9 g, 36.4 mmol, 1.00 *eq*) was dissolved in HOAc (70% in H_2O, 159 mL). The mixture was stirred at 100 °C for 1 hr. TLC (petroleum ether/ethyl acetate= 3/1, Rf = 0.04) indicated compound **6** was consumed completely and one new spot formed. The mixture was pour into water (300 mL), extracted with ethyl acetate (300 mL x 2), washed organic phase with saturate aq. $NaHCO_3$ (20 0mL x 2), concentrate in vacuum. The residue was purified by column chromatography (SiO_2,

petroleum ether/ethyl acetate=10/0 to 0/1). Compound **7** (10.0 g, 23.9 mmol, 65.7% yield, 95.0% purity) was obtained as a pale-yellow oil.

TLC: petroleum ether/ethyl acetate = 3/1, R_f = 0.04

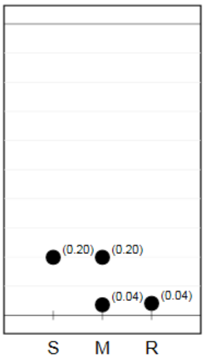

^{1}H NMR: ET38781-42-P1A1, 400 MHz, MeOD

δ ppm 7.92 (s, 1H), 7.67 (d, *J* = 9.2Hz, 1H), 7.47 (d, *J* = 7.2 Hz, 2H), 7.37 (t, *J* = 7.4 Hz, 2H), 7.31 (d, *J* = 7.2 Hz, 1H), 7.13 (s, 1H), 7.03 (dd, *J* = 8.8 Hz, 2.4 Hz, 1H), 5.21 (d, *J* = 3.2 Hz, 1H), 5.15 (s, 2H), 4.09-4.05 (m, 1H), 4.07 (t, *J* = 3.81 Hz, 1H), 4.02-3.99 (m, 2H), 3.97-3.95 (m, 1H), 3.83-3.81 (m, 4H), 3.80 (m, 1H)

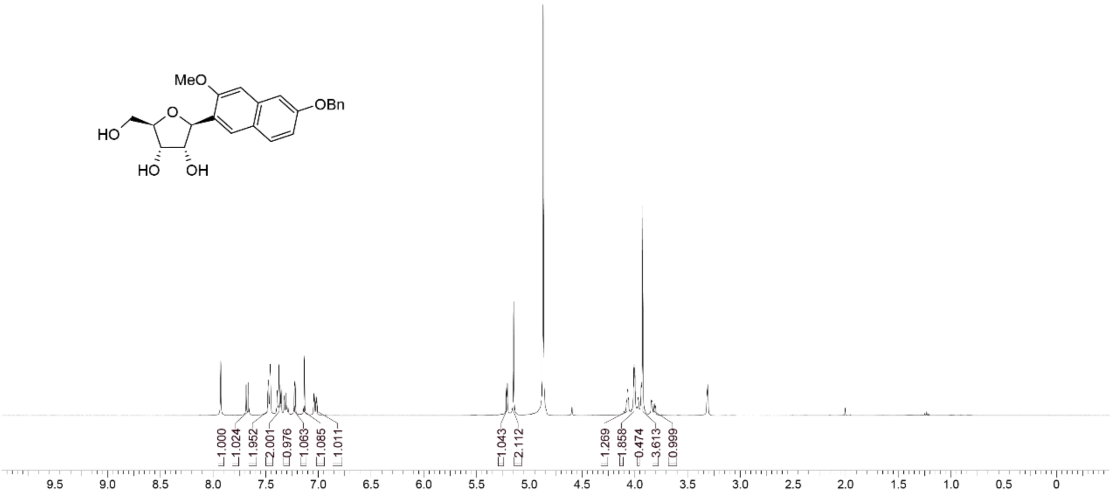

General procedure for preparation of compound 8

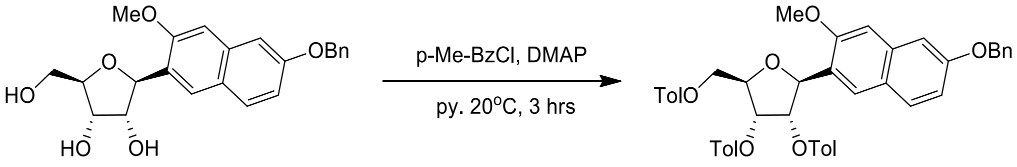

7

8

To a solution of compound **7** (12.0 g, 30.2 mmol, 1.00 *eq*) in Py (84.0 mL) was added DMAP (369 mg, 3.03 mmol, 0.10 *eq*), p-Me-BzCl (16.3 g, 105 mmol, 14.0 mL, 3.50 *eq*). The mixture was stirred at 20 °C for 3 hrs. TLC (petroleum ether/ethyl acetate= 0/1, Rf = 0.80) indicated compound **7** was consumed completely and one new spot

formed. The mixture was poured into water (500 mL) and solid precipitation. Filter and to give filter cake. Compound **8** (21.0 g, 27.9 mmol, 92.4% yield) as a white solid. TLC: petroleum ether/ethyl acetate = 0/1, R_f = 0.80

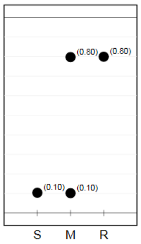

^{1}H NMR: ET38781-48-P1A1, 400 MHz, $CDCl_3$

δ ppm 8.01 (d, *J* = 8.0 Hz, 2H), 7.96 (d, *J* = 8.0 Hz, 2H), 7.90 (s, 1H), 7.77 (d, *J* = 8.0 Hz, 2H), 7.54-7.48 (m, 2H), 7.47-7.40 (m, 2H), 7.40-7.33 (m, 2H), 7.33-7.21 (m, 3H), 7.19 (d, *J* = 8.0 Hz, 2H), 7.16-7.08 (m, 3H), 7.07-6.95 (m, 2H), 5.91(dd, *J* = 5.2 Hz, *J* = 3.5 Hz, 1H), 5.7 9-5.76 (m, 2H), 5.65 (d, *J* = 3.0 Hz, 1H), 4.96 (dd, *J* = 12.2Hz, *J* = 2.9 Hz, 1H), 4.75 (m, 1H), 4.62 (dd, *J* = 12.2Hz, *J* = 3.9 Hz, 1H), 3.86(s, 3H), 2.43-2.36 (m, 9H),

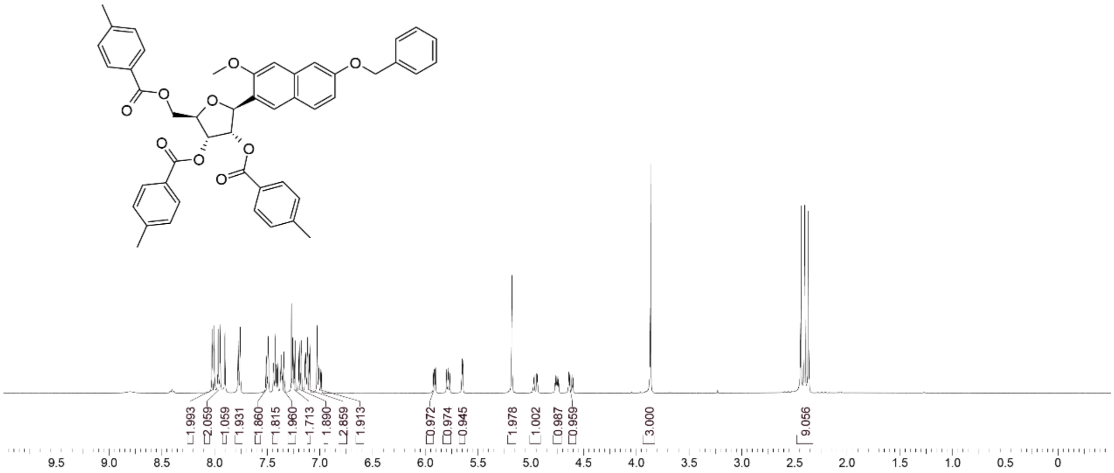

General procedure for preparation of compound 9

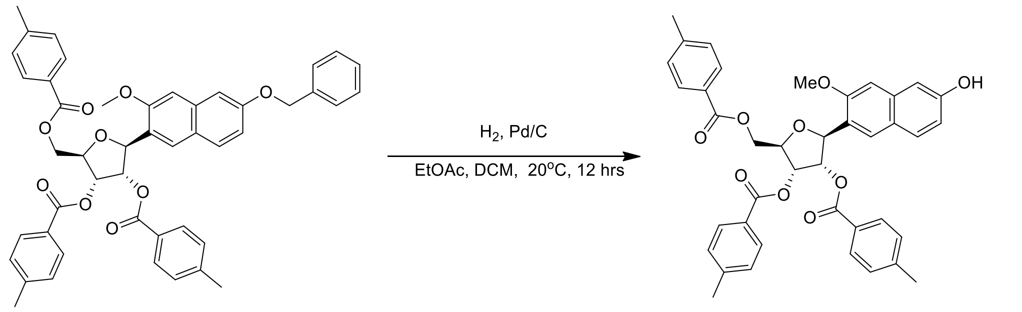

8

9

To a solution of Pd/C (5.00 g, 10% purity) in EtOAc (20 mL) was added compound **8**

(21.0 g, 27.9 mmol, 1.00 *eq*) in DCM (147 mL) under H_2 atmosphere. The suspension was degassed and purged with H_2 for 3 times. The mixture was stirred under H_2 (15 Psi) at 20 °C for 12 hrs. TLC (petroleum ether/ethyl acetate =3/1, production: R_f = 0.20) showed compound **8** was consumed completely. The reaction mixture filtered and concentrated under reduced pressure to give a residue. The residue was purified by column chromatography (SiO_2, petroleum ether/ethyl acetate = 50/1 to 2/1). Compound **9** (15.0 g, 22.7 mmol, 81.1% yield) was obtained as a white solid.

TLC: petroleum ether/ethyl acetate = 3/1, R_f = 0.20

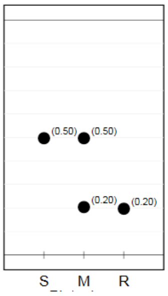

^{1}H NMR: ET38781-50-P1A1, 400 MHz, $CDCl_3$

δ ppm 7.92-7.85 (m, 4H), 7.74 (s, 1H), 7.68 (d, *J* = 8.0 Hz, 2H), 7.25-7.13 (m, 3H), 7.12–6.97 (m, 4H), 6.89 (d, *J* = 2.2 Hz, 1H), 6.68-6.80 (m, 2H), 5.86 (dd, *J* =5.0 Hz, *J* = 3.4 Hz, 1H), 5.71 (dd, *J* =7.7 Hz, *J* = 5.4 Hz, 2H), 5.54 (d, *J* = 3.2 Hz, 1H), 4.87 (dd, *J* = 12.1 Hz , *J* = 3.0 Hz, 1H), 4.68 (m, 1H), 4.55 (dd, *J* = 12.1 Hz, *J* = 4.2 Hz, 1H), 3.67 (s, 3H), 2.34 (s, 3H), 2.28 (d, *J* = 5.5 Hz, 6H)

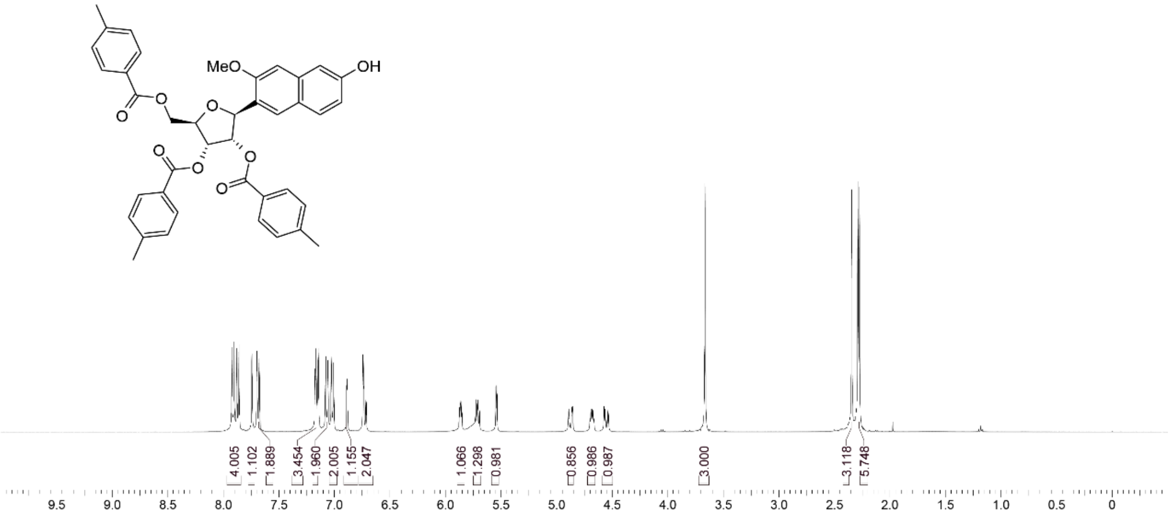

General procedure for preparation of compound 10

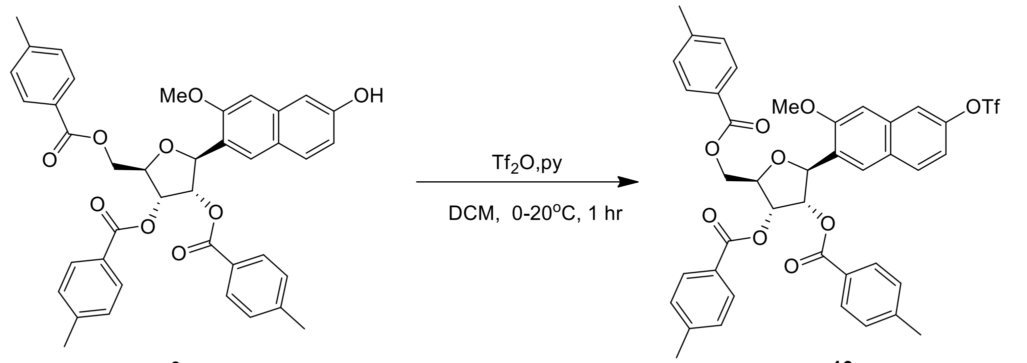

9 **10**

To a solution compound **9** (5.00 g, 7.57 mmol, 1.00 *eq*) and Py (3.59 g, 45.4 mmol, 3.66 mL, 6.00 *eq*) in DCM (30 mL) was added Tf_2O (3.20 g, 11.3 mmol, 1.87 mL, 1.50 *eq*) dropwise at 0 °C under N_2. The mixture was stirred at 20 °C for 1 hr. TLC (petroleum ether/ethyl acetate = 3/1, R_f = 0.70) showed the reaction was complete. The reaction mixture was poured into water (300 mL), extracted with DCM (300 mL), concentrated organic phase in vacuum to give crude product. The crude product was purified by silica gel chromatography (SiO_2, petroleum ether/ethyl acetate = 20/1 to 3/1). Compound **10** (5.50 g, 6.94 mmol, 91.6% yield) was obtained as a white solid.

TLC: petroleum ether/ethyl acetate = 3/1, R_f = 0.70

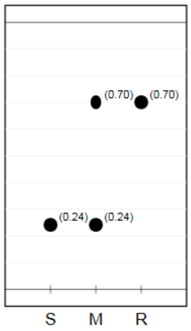

^{1}H NMR: ET38781-52-P1A1, 400 MHz, $CDCl_3$

δ ppm 7.94-7.86 (m, 5H), 7.69 (d, *J* =8 .0 Hz, 2H), 7.54 (d, *J* =2.2 Hz, 1H), 7.32 (d, *J* =9.0 Hz, 1H), 7.23-7.14 (m, 2H),7.12-7.07 (m, 1H), 7.09 (d, *J* = 8.0 Hz, 1H), 7.06-6.97 (m, 4H), 5.82 (dd, *J* = 5.2 Hz, *J* = 3.4 Hz, 1H), 5.67 (dd, *J* = 7.6 Hz, *J* = 5.4 Hz, 1H), 5.58 (d, *J* = 2.7 Hz, 1H), 4.94 (dd, *J* = 12.2 Hz, *J* = 2.8 Hz, 1H), 4.75-4.64 (m, 1H), 4.51 (dd, *J* = 12.3 Hz, *J* = 3.69 Hz, 1H), 3.78 (s, 3H), 2.35-2.28 (m, 9H)

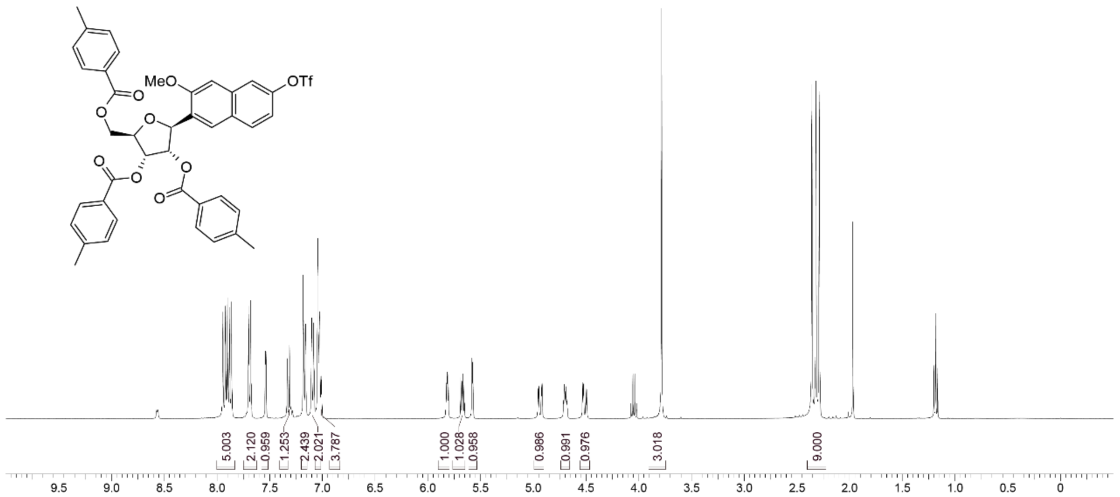

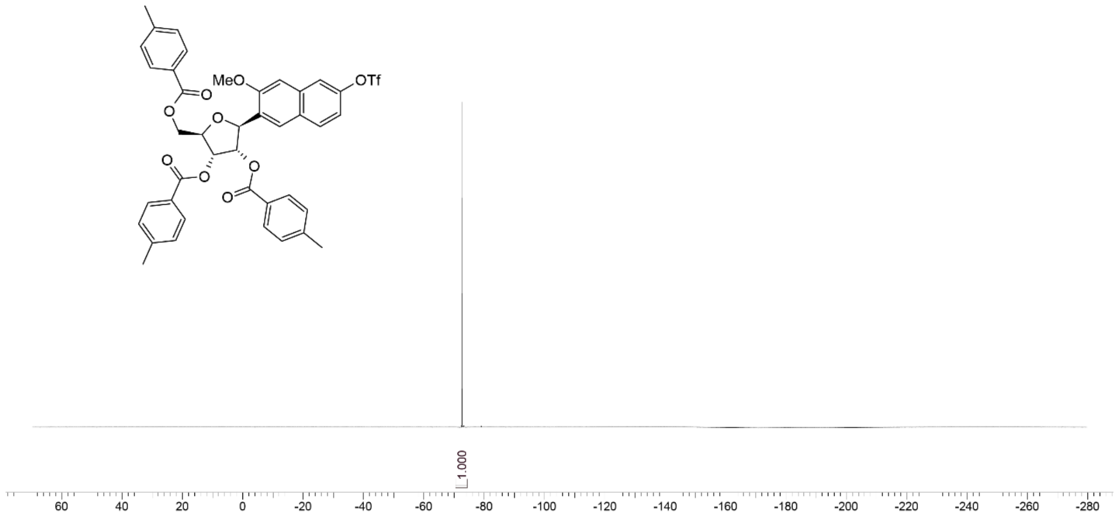

General procedure for preparation of compound 12

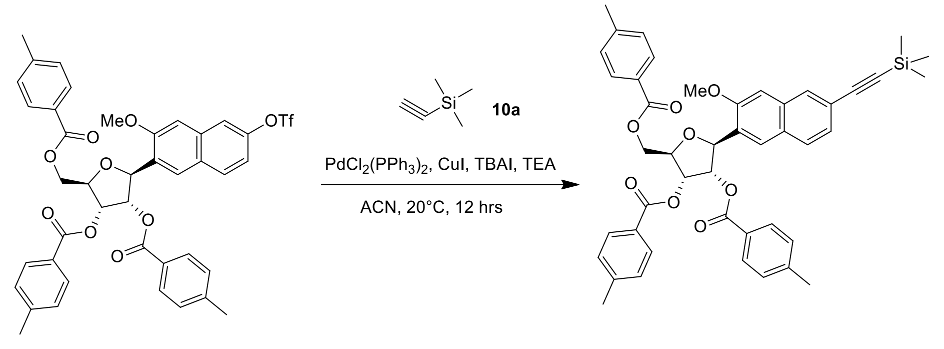

10 **11**

To a solution compound **10** (2.75 g, 3.53 mmol, 1.00 *eq*) and compound **10a** (1.04 g, 10.5 mmol, 1.47 mL, 3.00 *eq*) in ACN (80.0mL) was added TEA (2.14 g, 21.2 mmol, 2.95 mL, 6.00 *eq*), $PdCl_2(PPh_3)_2$ (743 mg, 1.06 mmol, 0.30 *eq*)，TBAI (3.91 g, 10.59 mmol, 3.00 *eq*) at 20 °C under N_2. Then, CuI (67.2 mg, 353 umol, 0.10 *eq*) was added and the reaction mixture was degassed with N_2 three times. The mixture was stirred at 20 °C for 12 hrs. TLC (petroleum ether/ethyl acetate = 2/1, R_f = 0.55) indicated

compound **10** was consumed completely and a new spot formed. Combine two batches. The reaction mixture was poured into water (300 mL), extracted with ethyl acetate (200mL x 2), concentrated in vacuum to give residue. The residue was purified by column chromatography (SiO_2, petroleum ether/ethyl acetate=100/1 to 3/1). Compound **11** (4.30 g, 5.80 mmol, 82.1% yield) was brown oil.

TLC: petroleum ether/ethyl acetate = 2/1, R_f = 0.55

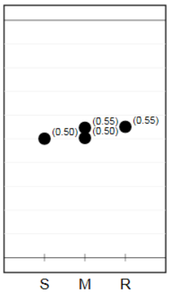

^{1}H NMR: ET38781-53-P1A1, 400 MHz, $CDCl_3$

δ ppm 8.01-7.94 (m, 5H), 7.88 (s, 1H), 7.76 (d, *J* = 8.0 Hz, 2H), 7.37-7.32 (m, 1H), 7.29 (m, 1H), 7.27-7.26 (m, 2H), 7.18 (d, *J* =8.0 Hz, 2H), 7.11 (d, *J* =8.0 Hz, 2H), 7.05 (s, 1H), 5.91 (dd, *J* = 5.2 Hz, *J* = 3.4 Hz, 1H), 5.76 (dd, *J* = 7.7 Hz, *J* = 5.4 Hz, 1H), 5.66 (d, *J* = 3.0 Hz, 1H), 4.97 (dd, *J* = 12.2 Hz, *J* = 2.8 Hz, 1H), 4.79-4.72 (m, 1H), 4.61 (dd, *J* = 12.2 Hz , *J* = 3.7Hz, 1H), 3.86 (s, 3H), 2.44-2.36 (m, 9H), 0.29 (s, 9H)

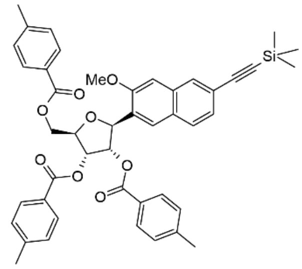

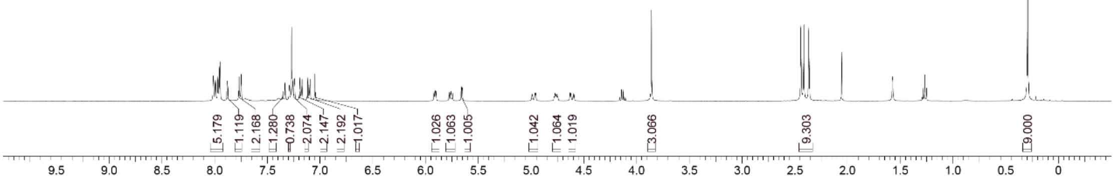

General procedure for preparation of compound 13

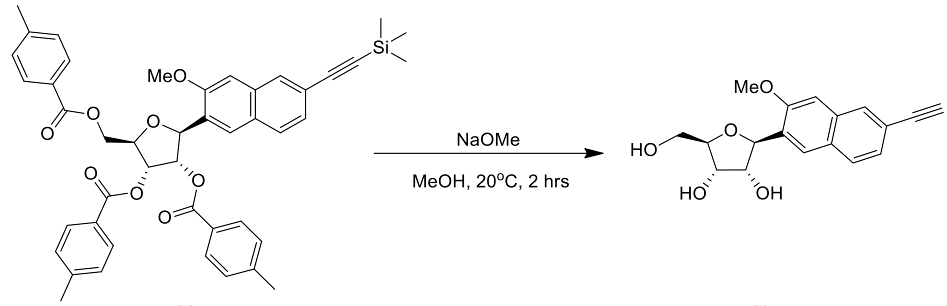

To a solution of compound **11** (3.90 g, 5.26 mmol, 1.00 *eq*) in DCM (20.0 mL) was added NaOMe (947 mg, 5.26 mmol, 30% purity, 1.00 *eq*) in MeOH (5.00 mL). The mixture was stirred at 20 °C for 2 hrs. LCMS (ET38781-57-P1A1, RT = 1.100 min, reaction solution) showed compound **11** was consumed completely and one main peak with desired m/z was detected. The mixture was poured into saturate aq.NH_4Cl (200 mL), extracted withDCM (100 mL x 2), concentrated organic phase in vacuum to give a residue. The residue was purified by prep-HPLC (neutral condition, column: Agela DuraShell C18 250 x 70mm x 10um; mobile phase: [water (10 mM NH_4HCO_3)-ACN]; B%: 22%-52%, 20 min). Compound **12** (1.00 g, 3.18 mmol, 60.4% yield) was obtained as a brown solid.

^{1}H NMR: ET38781-57-P1A1, 400 MHz, MeOD

δ ppm 8.05 (s, 1H), 7.91 (s, 1H), 7.74 (d, *J* = 8.4 Hz, 1H), 7.33 (dd, *J* = 8.4 Hz, *J* = 1.2 Hz, 1H), 7.23 (s, 1H), 5.24 (d, *J* = 2.8 Hz, 1H), 4.04-4.00 (m, 7H), 3.99-3.86 (m, 1H), 3.52 (s, 1H)

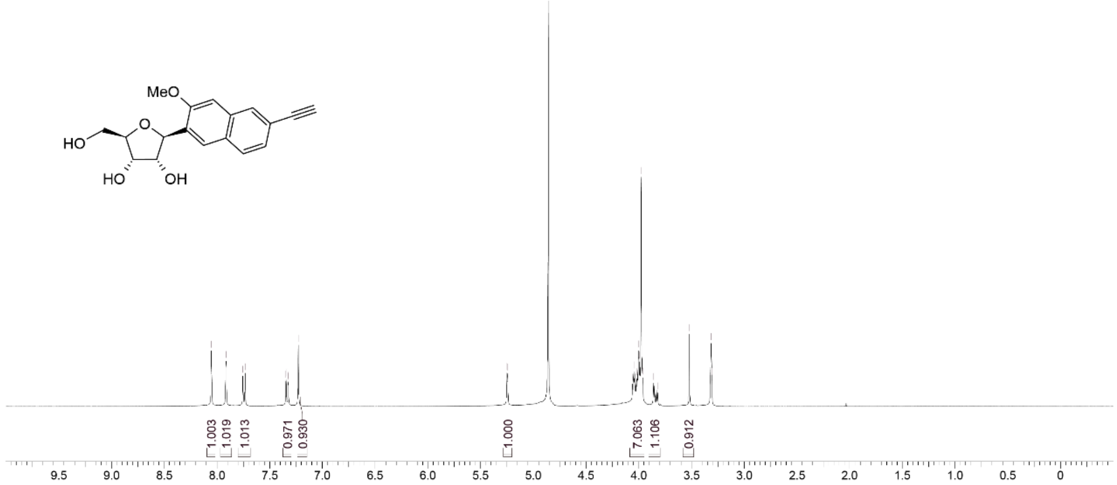

General procedure for preparation of compound 12a

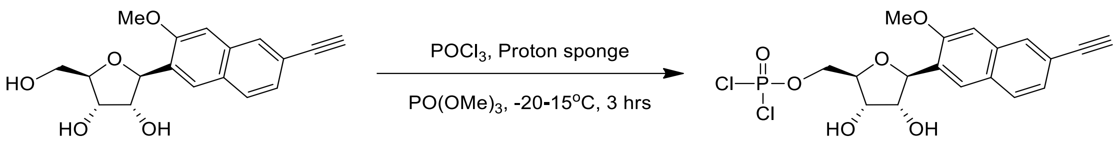

12 **12a**

Compound **12** (0.80 g, 2.55 mmol, 1.00 *eq*), proton sponge (545 mg, 2.55 mmol, 1.00 *eq*) was dissolved in CH_3CN (20 mL x 3), evaporated to dryness. To a solution of compound **12** (0.8 g, 2.55 mmol, 1.00 *eq*), proton sponge (545 mg, 2.55 mmol, 1.00 *eq*) in $PO(OMe)_3$ (8.00 mL) was added $POCl_3$ (507 mg, 3.31 mmol, 1.30 *eq*) at -20 °C. The mixture was stirred at 15 °C for 3 hrs. LCMS (ET38781-62-P1A1, RT = 1.258 min, reaction solution) showed compound **12** was consumed completely and one main peak with desired m/z was detected. Compound **12a** (1.10 g, crude) was brown liquid was used into the next step without further purification.

General procedure for preparation of compound 13

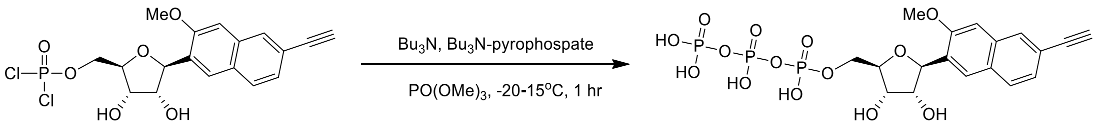

12a **13**

To a solution of compound **12a** (1.10 g, 2.55 mmol, 1.00 *eq*) in $PO(OMe)_3$ (8.00 mL) was added Bu_3N (2.84 g, 15.31 mmol, 6.00 *eq*) and Bu_3N- pyrophospate (0.6 M, 21.2 mL, 5.00 *eq*) at -20 °C. The mixture was stirred at 15 °C for 1 hr. LCMS (ET38781-64-P1A1, RT = 1.220 min, reaction solution) showed compound **12a** was consumed completely and one main peak with desired mass was detected. Added 1 M TEAB to pH 7, the solution was diluted with H_2O 500 mL and extracted with MTBE (500 mL x 3). The reaction mixture purified by a DEAE Sephadex column with an elution gradient of 0 to 1.00 M TEAB, evaporated to obtain a colorless oil. The colorless oil was purified by prep-HPLC (neutral condition: column: Agela DuraShell C18 25 x 70 mm x10 um; mobile phase: [water (10 mM NH_4HCO_3)-ACN]; B%: 1%-13%, 20 min). Compound **13** (0.70 g, 1.26 mmol, 49.5% yield, TEA) was obtained as a white solid.

^{1}H NMR: ET38781-64-P1A1, 400 MHz, D_2O

δ ppm 8.02 (s, 1H), 7.93 (s, 1H), 7.86 (d, *J* = 8.4 Hz, 1H), 7.42 (d, *J* = 8.0 Hz, 1H), 7.25 (s, 1H), 5.23 (d, *J* = 2.4 Hz, 1H), 4.26-4.19 (m, 5H), 3.90 (s, 3H), 3.50 (s, 1H)

^{31}P NMR: ET38781-64-P1A1, 162 MHz, D_2O

δ ppm -9.97 (d, *J* = 19.6 Hz, 1P), -11.21 (d, *J* = 19.6 Hz, 1P), -23.04 (t, *J* = 20.7 Hz, 1P)

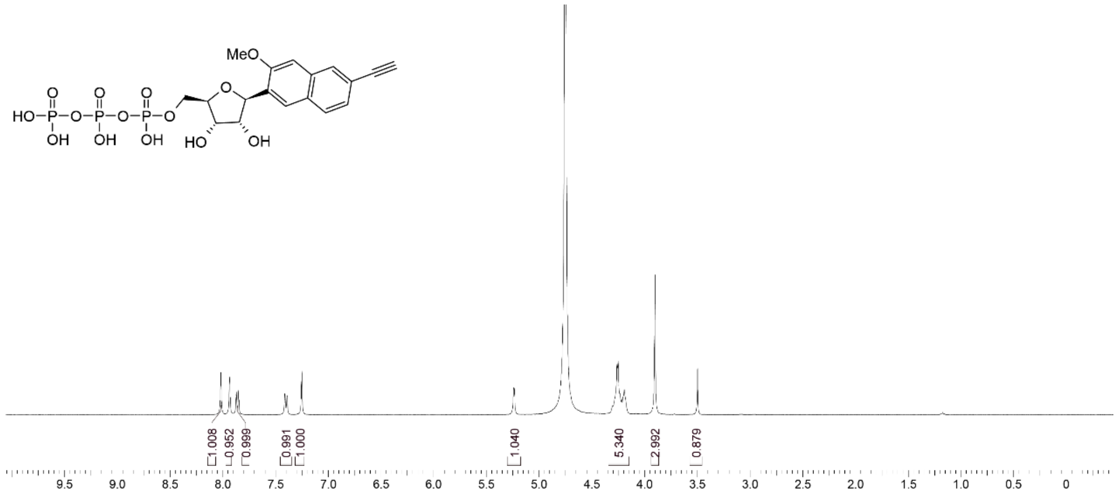

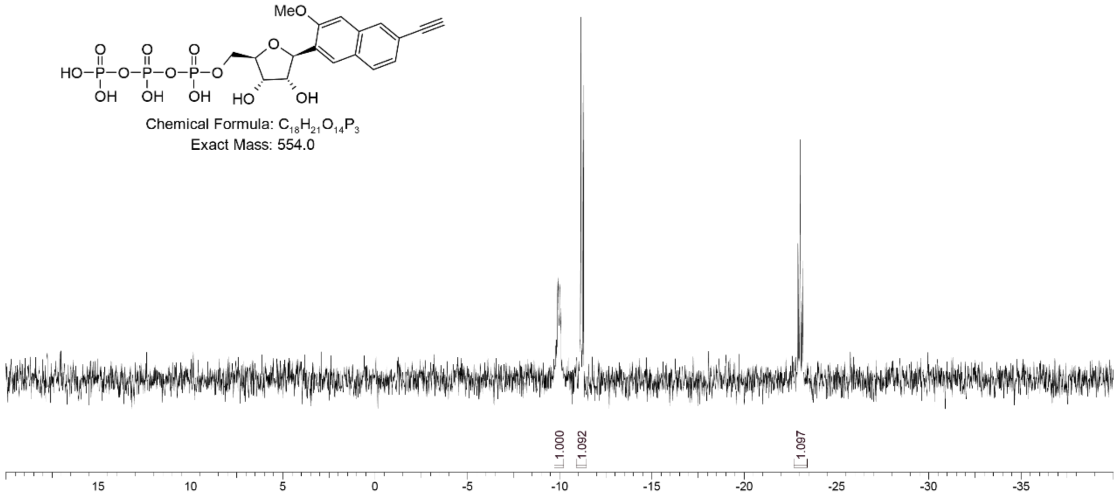

General procedure for preparation of rNaM^{CO}TP

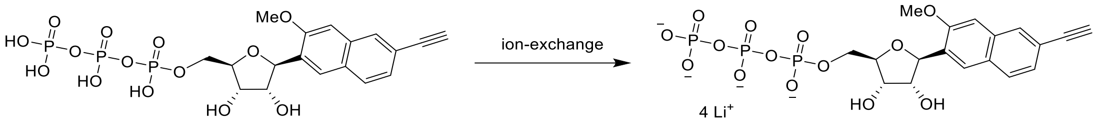

13 **NaMTP-CO**

A mixture of compound **13** (0.70 g, 1.26 mmol, 1.00 *eq*, TEA) in H_2O (5 mL), then through Li^+ resin at 25 °C. The target compound was showed spot at TLC, when the spot disappeared, stop added water. Then lyophilized the water. NaMTP-CO (0.5 g, 890.93 μmol, 70.5% yield, Li^+) was obtained as a white solid.

^{1}H NMR: ET38781-65-P1C1, 400 MHz, D_2O

δ ppm 8.04 (s, 1H), 7.95 (s, 1H), 7.88 (d, J = 8.4 Hz, 1H), 7.42 (dd, J = 8.4 Hz, 1.2 Hz, 1H), 7.26 (s, 1H), 5.26 (d, J = 2.8 Hz, 1H), 4.36-4.22 (m, 5H), 3.93 (s, 3H), 3.54 (s, 1H)

^{31}P NMR: ET38781-65-P1C1, 162 MHz, D_2O

δ ppm -8.64--7.83 (m, 1P), -10.93 (d, J = 17.4 Hz, 1P), -21.99 (t, J = 18.5 Hz, 1P).

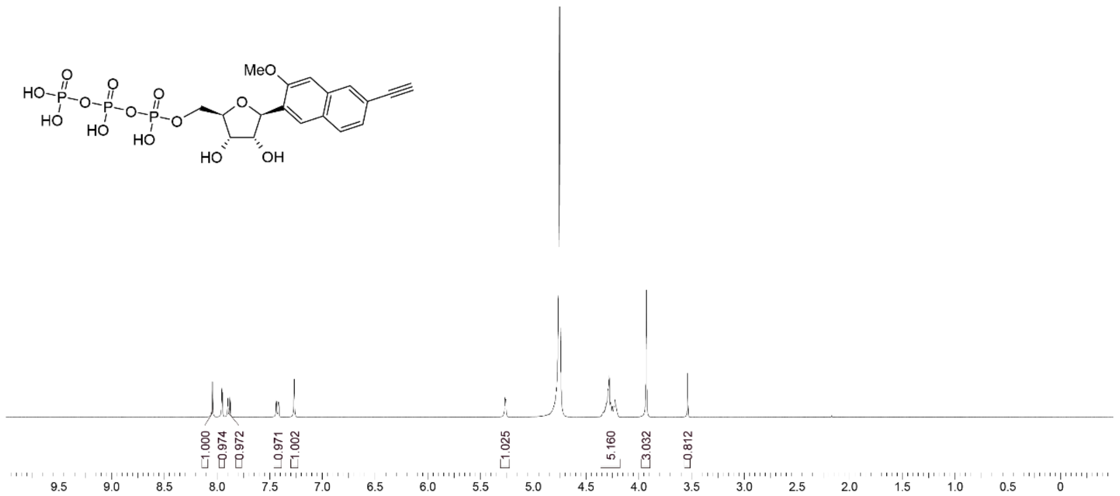
MeO
HO
OH
1.000
0.974
0.972
0.971
1.002
1.025
5.160
3.032
0.812
9.5 9.0 8.5 8.0 7.5 7.0 6.5 6.0 5.5 5.0 4.5 4.0 3.5 3.0 2.5 2.0 1.5 1.0 0.5 0

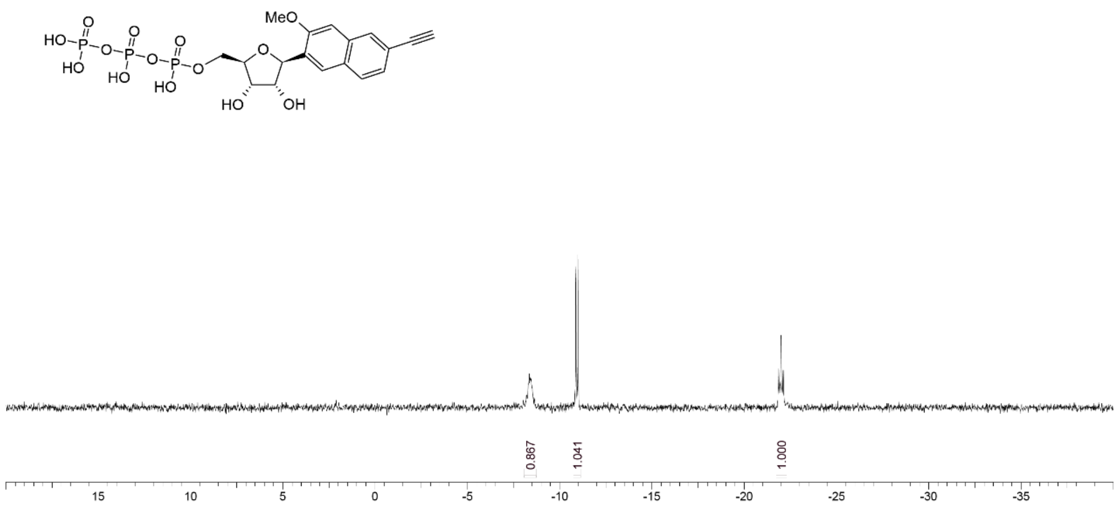
MeO
HO
OH
0.867
1.041
1.000
15 10 5 0 -5 -10 -15 -20 -25 -30 -35

Generating all-atom 3D models with unbiased MD simulations

The conformational pools for *apo*-form T99, T89 and T77 RNAs were generated with unbiased all-atom MD simulations in explicit solvents, which were then used to screen out a best-fit model consistent with the experimental SAXS data. Considering that the RNAs studied here are relatively large for all-atom MD simulations and the conformational landscape of RNA is generally rather rugged, to accelerate exploration of the conformational spaces with MD simulations, we constructed different initial structural configurations for the respective RNAs. Such strategy has been successfully applied to study the complex biological processes and to estimate transition rates between different states when combined with Markov state models analysis[1]. It should be noted that our MD simulation herein is to search the best structural model matching experimental observations, but not to directly reconstruct the conformational energy landscape of the RNAs. The latter requires exhaustive sampling, which could be a huge computational burden for large RNA.

Preparation of initial structural configuration for MD simulations

The tRNA-bound T99 crystal structure model (PDB code: 6UFM) was directly used to prepare the starting models of T99, T89 and T77 by removing the tRNA or the extra nucleotide fragments.

During the simulation for *apo*-T99 at high Mg^{2+}, we observed that the Specifier loop of stem I spontaneously dissociates from the S-turn of stem II, which we referred as undocked state of T99 (**Supplementary Fig. 9a-b**). The configuration of T99 in undocked state was used as the template to build initial structural models for T77 and T89. In Parallel, we employed homology modeling using FARFAR2[2] Program with T99 as template to construct another sets of undocked configurations with different mutual orientation between Stem I and Stem II. During the simulation for *apo*-T77 at high

Mg^{2+}, Stem I was found to spontaneously stack on Stem II, resulting in a thermodynamically favorable and extended conformation (**Supplementary Fig. 9c-e**). SAXS data suggested that the conformation of *apo*-T77 is less affected by Mg^{2+}. smFRET data suggested that stem IIA/B in T99 is partially unfolded at low Mg^{2+}. According to the observations of SAXS and smFRET and simulations on T77, we used FARFAR2[2] to constructed an initial model for T99 at low Mg^{2+} with the last 10 nucleotides (90 to 99) being in the single-stranded state combined with two different stacked states including stem I being stacked on stem II or stem IIA stacking on stem II. Similarly, we also utilized FARFAR2[2] to construct an initial configuration for the simulations of T89 at low Mg^{2+}, with stem I being stacked on stem II or with stem II stacking on stem II. A simple description of the constructs with different initial configurations for MD simulation is summarized in **Supplementary Table 7**.

Simulation settings

The RNA was parameterized using parmbsc0+χOL with van der Waals radii correction to phosphate oxygen atoms[3-7], and water was parameterized using the 4-point optimal point-charge (OPC) model[8]. The ion parameters developed by the Merz group for OPC water were employed in our simulations[9,10]. OPC water models have been shown to better reproduce the hydration shell of biomolecules, which is essential to avoid overly compact conformation[11]. Two Mg^{2+} ionic conditions were taken into consideration: (1) none (represents lower Mg^{2+} concentration); (2) the amount of Mg^{2+} needed to neutralize each system (represents higher Mg^{2+} concentration). To avoid potentially artificial effect induced by newly added Mg^{2+} ions, the ions were initially placed at positions which are at least 6 Å distal to RNA surface, since Mg^{2+} ions can tightly bind to the negatively charged phosphate moiety with a residence time (>> μs) much longer than the timescale accessible to MD simulation in current. For all systems, NaCl was

added to neutralize the system and yield an ionic concentration of 150 mM. The rhombic dodecahedron simulation boxes contain approximately 150,000 to 200,000 atoms (**Supplementary Table 7**).

All MD simulations were conducted using GROMACS 2022.3[12]. All systems were firstly minimized with positional restraints on non-hydrogen atoms of RNA along with Mg^{2+}, followed by second minimization with positional restraints on Mg^{2+} and a step-wise heating from 10 K to 310 K with positional restraints on non-hydrogen atoms of RNA and Mg^{2+}. Upon the completion of 0.6 ns lengthed heating phase, the newly added Mg^{2+} ion is expected to form stable hydration configuration with 6 molecules of water in first coordination sphere. Then the positional restraint on Mg^{2+} ions were removed in the following equilibrations, allowing them to freely diffuse. Two sequential NPT simulations (T = 310 K and P = 1 bar) were then carried out in with positional restraint on non-hydrogen atoms of RNA. During the heating as well as NPT phases, the strength of positional restraints imposed on RNA were gradually reduced to further relax RNA. Finally, for each system, the production run was carried out in NPT ensemble without any restraints for at least 100 ns. In production phase, the coordinates of system were saved every 0.1 ns for the following structural analysis. Taken together, we performed unbiased simulation with different initial structures instead of experimental data-driven or enhanced sampling simulation to generate conformational pool, as mentioned above. The temperature and pressure were maintained by the velocity-rescaling thermostat, and the Parrinello-Rahman barostat respectively[13,14]. The covalent bonds involved by hydrogen atoms were constrained using the LINCS algorithm[15], allowing an integration time step of 2 fs. Periodic boundary conditions were applied in three directions. van der Waals and short-range electrostatic interactions were calculated at a cutoff distance of 10 Å, whereas long-range electrostatic interactions were computed by the particle mesh

Ewald method[16].

After simulation, the SAXS spectra for each conformer from the initial conformation pool were back-calculated using CRYSOL[17]. Following the philosophy of minimum ensemble size, single conformer to best match our experimental observables was selected, which can be approximately regarded as the centroid structure of an ensemble corresponding to the respective macrostate.

Visualization of conformation change of T99

We employed MD simulation with structure-based model (SMOG)[18,19] to generate continuous structural movement between two states, to illustrate the conformational change of T99 RNA induced by Mg^{2+} as well as its cognate tRNA. Distinct from physics-based model (we used to generate conformation pool which was screen against SAXS data) that describes the interactions with general rule, the structure-based model explicitly differentiates non-native interaction from native interactions that are observed in experimentally-determined structure. The native contact interations are described by attractive Lennard-Jones potentials, whereas the non-native contact are disfavored and described by exclude volume. As a consequence, the energetic landscape undermined by structure-based model is thus much smoother than that observed in physics-based model. Taking advantage of minimal frustration nature of structure-based model, we successfully “modeled” the conformational change with very limited computational cost (within 1 core.hour for each system).

More specifically, we used the final state of the each process to construct the respective Hamiltonian with SMOG server[18]. The initial and final state structures of each process were taken from physics-based MD simulations, which match our experimental observables on different conditions (i.e. Mg^{2+} or tRNA). More specifically, the initial and final state structures of Mg^{2+}-induced folding system are the representative

structural models of *apo*-T99 satisfying experimental data on low and high Mg^{2+} concertration, respectively. The initial structure of tRNA-induced folding system was taken from final state structure of Mg^{2+}-induced folding system; whereas its final state structure was taken from the MD-relaxed model of *holo*-T99. Therefore, the simulation of conformational change will be efficientely guided by the information encoded in the final state. Following the procedure in our previous work[20], we performed structure-based MD simulation with GROMACS 2022.3[12]. The resultant trajectories were utilized to generated movies with VMD 1.9.3[21] and VideoMach 5.15.1 program.

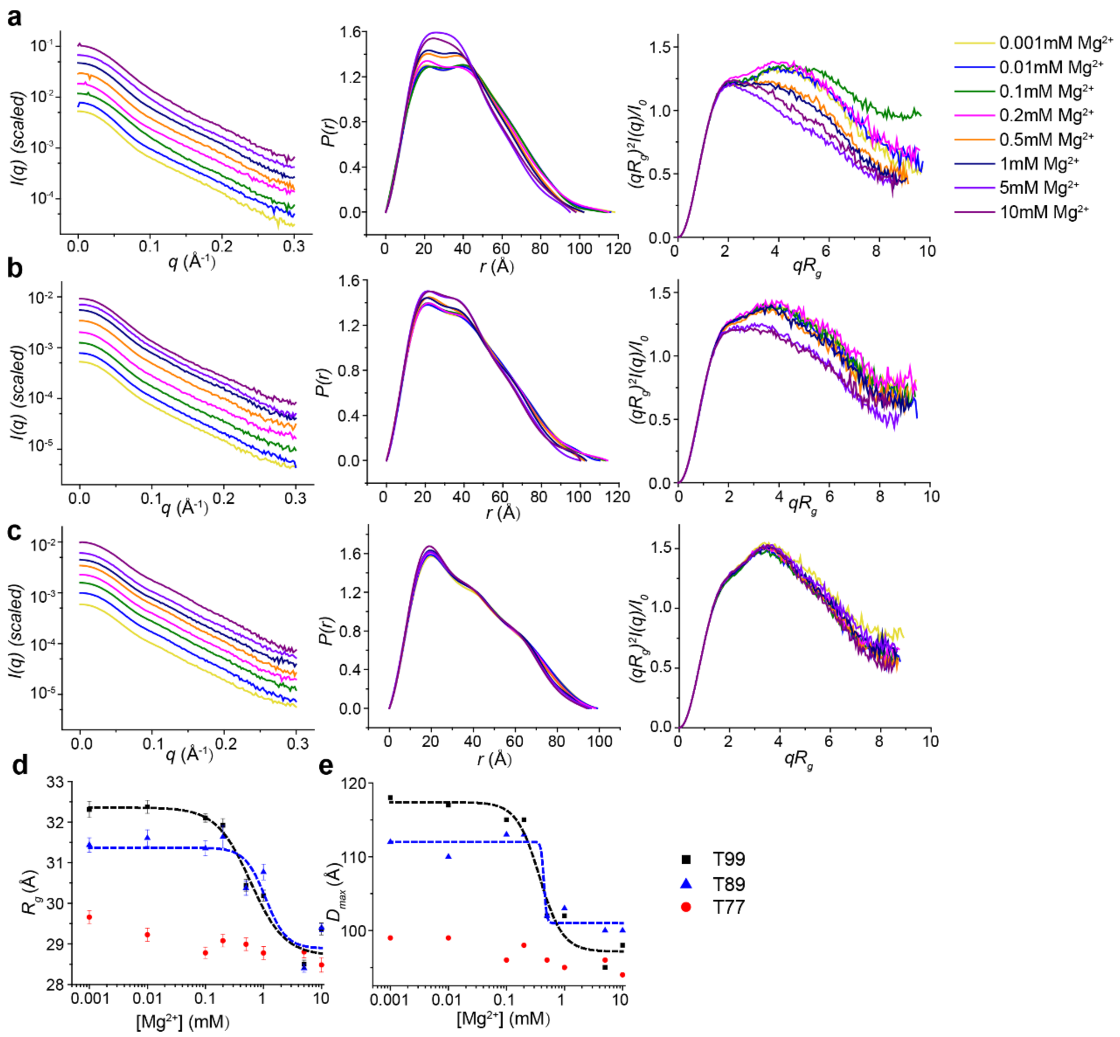

Supplementary Figure 1. **The effects of Mg^{2+} on the folding of T99, T89 and T77 analyzed by SAXS. (a-c)** The scattering curves (left)**,** normalized PDDFs (middle) and the dimensionless Kratky plots (right) for the T99 (**a**), T89 (**b**) and T77 (**c**) RNAs across various Mg^{2+} concentrations ranging from 0.001 to 10 mM. The color codes are indicated on the upper right and are the same for **a-c.** (**d-e**) Plots of R_g (**d**) and D_{max} (**e**) derived from PDDFs as a function of Mg^{2+} for T77 (red circle), T89 (blue triangle) and T99 (black square). Each data point in **d** represents an independent experiment (n = 1) and the error bars are propagated uncertainties calculated by GNOM. Source data are provided as a Source Data file.

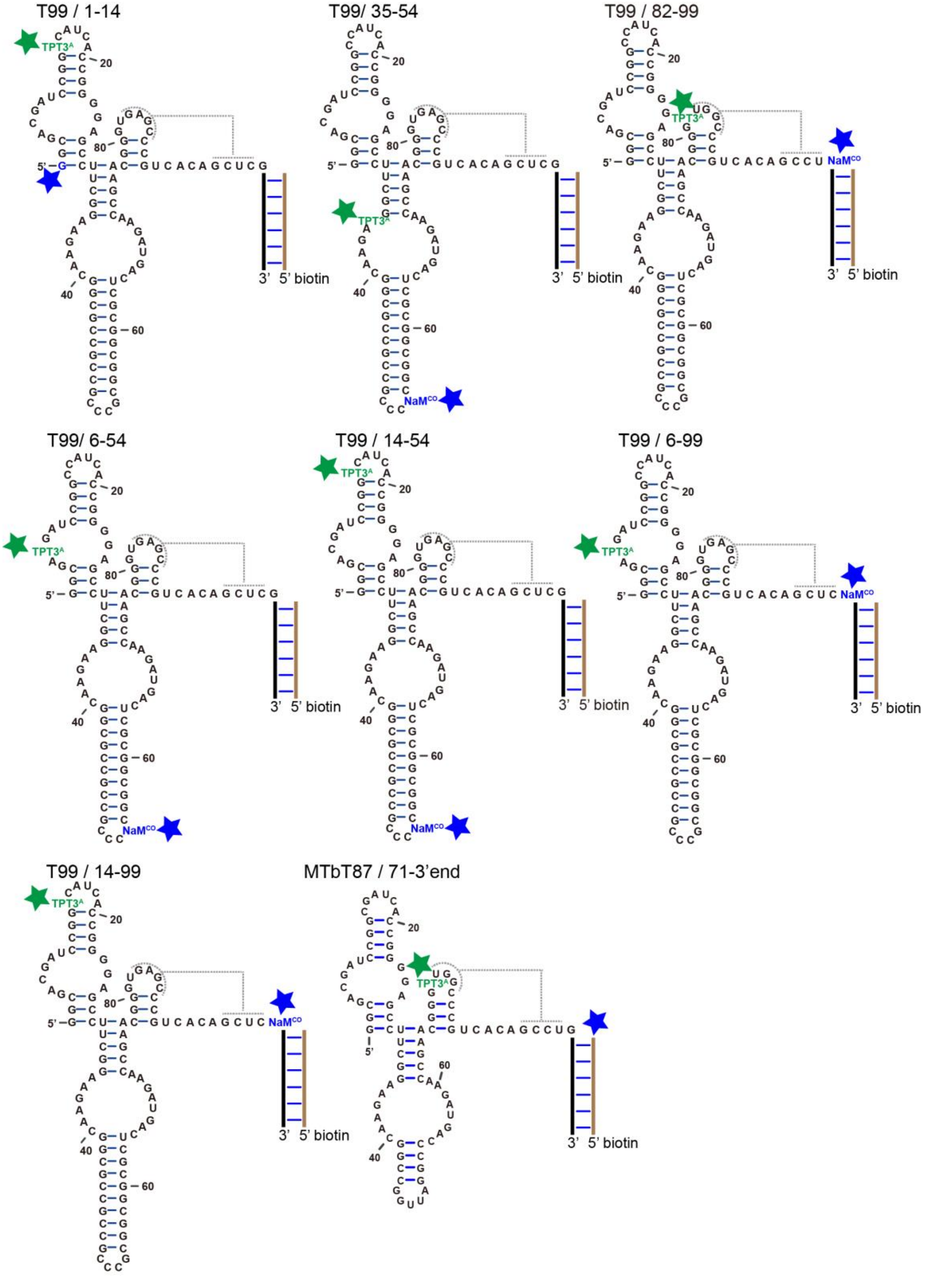

Supplementary Figure 2. **The sequences and secondary structures of *N.far* T99 and *M. tub* T87 constructs used in the smFRET experiments.** The Cy3 and Cy5 labeling sites are represented as green and blue stars, respectively. The RNA extension at the 3' end of T99 and the 5'-biotin labeled DNA oligonucleotides are represented with black and brown lines, respectively.

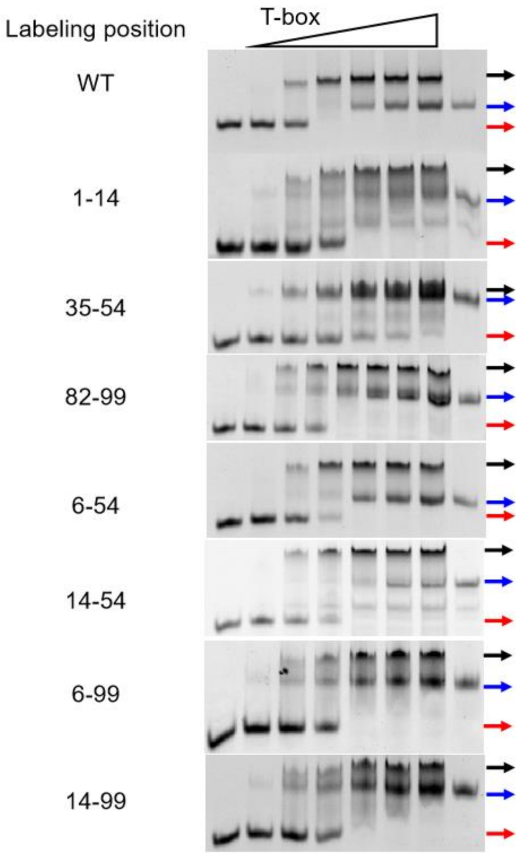

Supplementary Figure 3. **UBP-based fluorophore labeling have a minor effect on the binding between T-box and tRNA.** EMSA assays were performed for all of the fluorophore labeled T-box RNAs by varying the T-box concentrations (the ratio for [T-box]:[tRNA] is 0:1, 0.1:1, 0.5:1, 1:1, 2:1, 2.5:1, 3:1, 1:0). The red, blue and black arrows represent the tRNA, T-box and T-box/tRNA complex, respectively. All of the experiments were conducted on independent triplicates (n = 3). Source data are provided as a Source Data file.

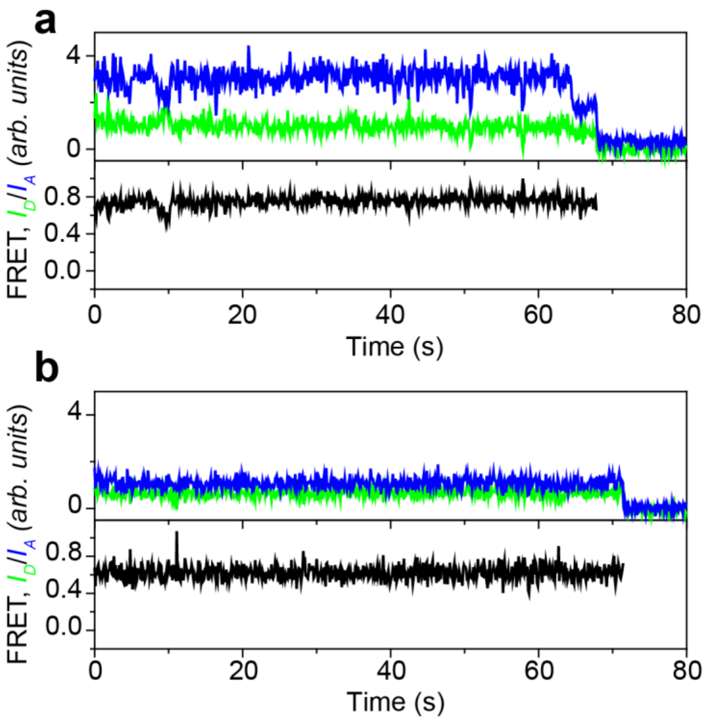

Supplementary Figure 4. **Additional representative smFRET traces for T99/1-14 in 0 mM Mg^{2+} (a) and 20 mM Mg^{2+} (b) in the absence of tRNA.** Source data are provided as a Source Data file.

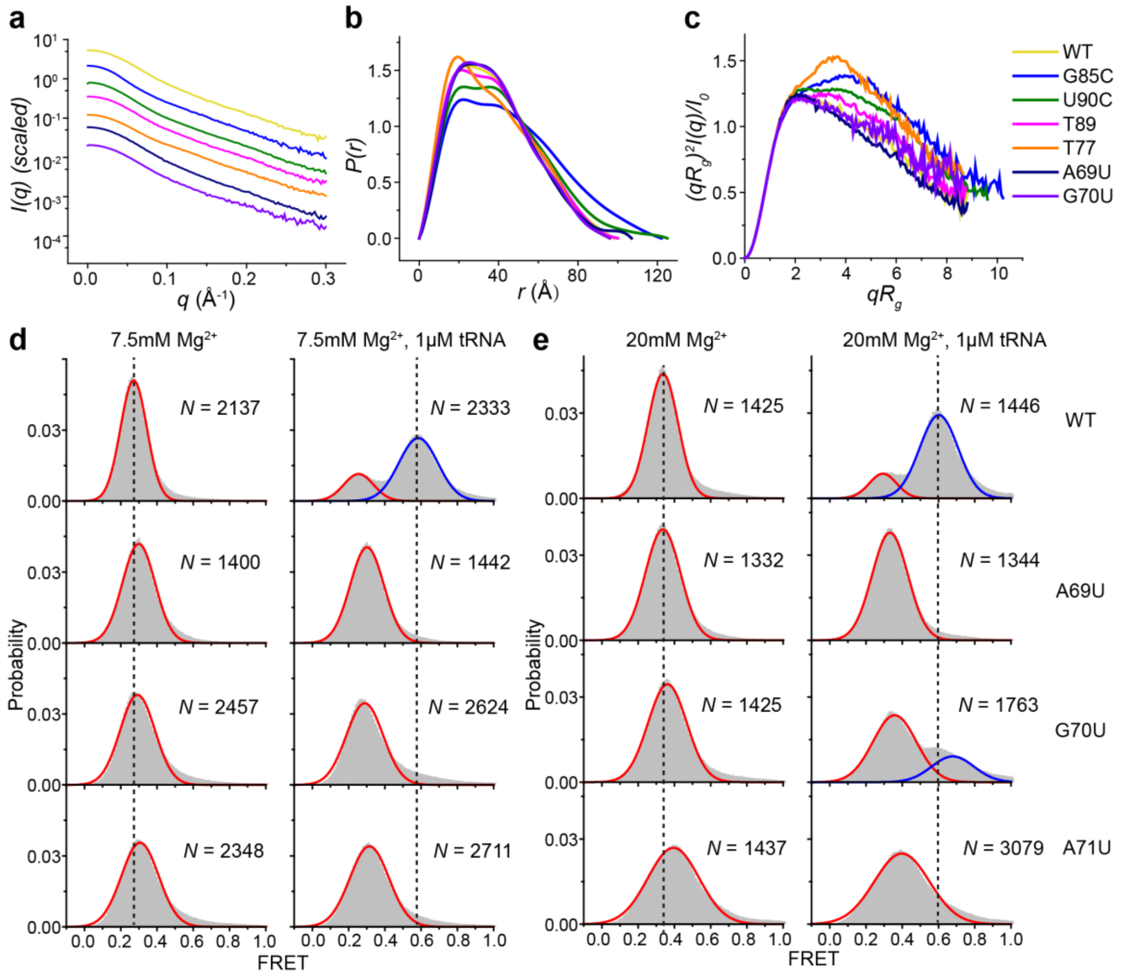

Supplementary Figure 5. **The effects of tertiary interaction mutants on the folding and structural dynamics of T99.** (**a-c**) The scattering curves **(a),** normalized PDDFs (**b**) and the dimensionless Kratky plots (**c**) for the T99 tertiary interaction mutants in 5 mM Mg^{2+}. The color codes are indicated on the right and the same for **c-e**. **(d-e)** FRET histograms for T99 WT and S-turn motif mutants (A69U, G70U, A71U) in the absence or presence of 1 μM tRNA supplemented with 7.5 mM Mg^{2+} (**d**) or 20 mM Mg^{2+} (**e**). *N* denotes the total number of traces to generate histograms from three independent experiments (n = 3). Source data are provided as a Source Data file.

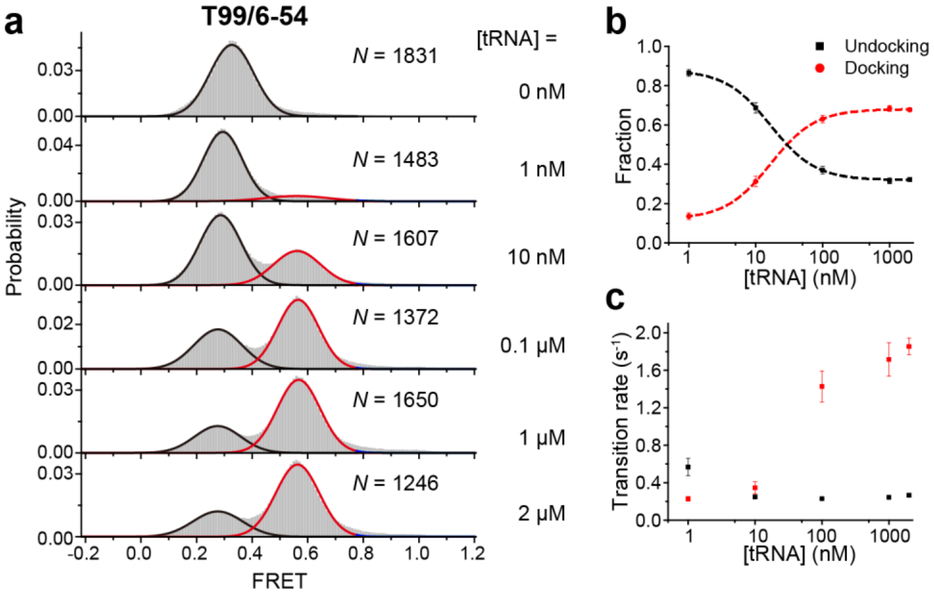

Supplementary Figure 6. **Binding of tRNA induced the docking between stems I and II. (a)** FRET histograms for T99/6-54 in the presence of varying concentrations of tRNA. *N* denotes the total number of traces to generate histograms from three independent experiments. The tRNA concentrations were indicated on the right. Histograms were well fitted with the Gaussian peaks, shown in black and red for the low- and middle-FRET states, respectively. (**b**) Fractional population of FRET states from two-state fitting to FRET histograms in **a** were plotted as a function of tRNA concentration and fitted using the Hill1 equation for T99/6-54. (**c**) Transition rates were plotted as a function of tRNA concentration. Data are presented as mean ± SEM from three independent experiments (n = 3) in **b-c**. Source data are provided as a Source Data file.

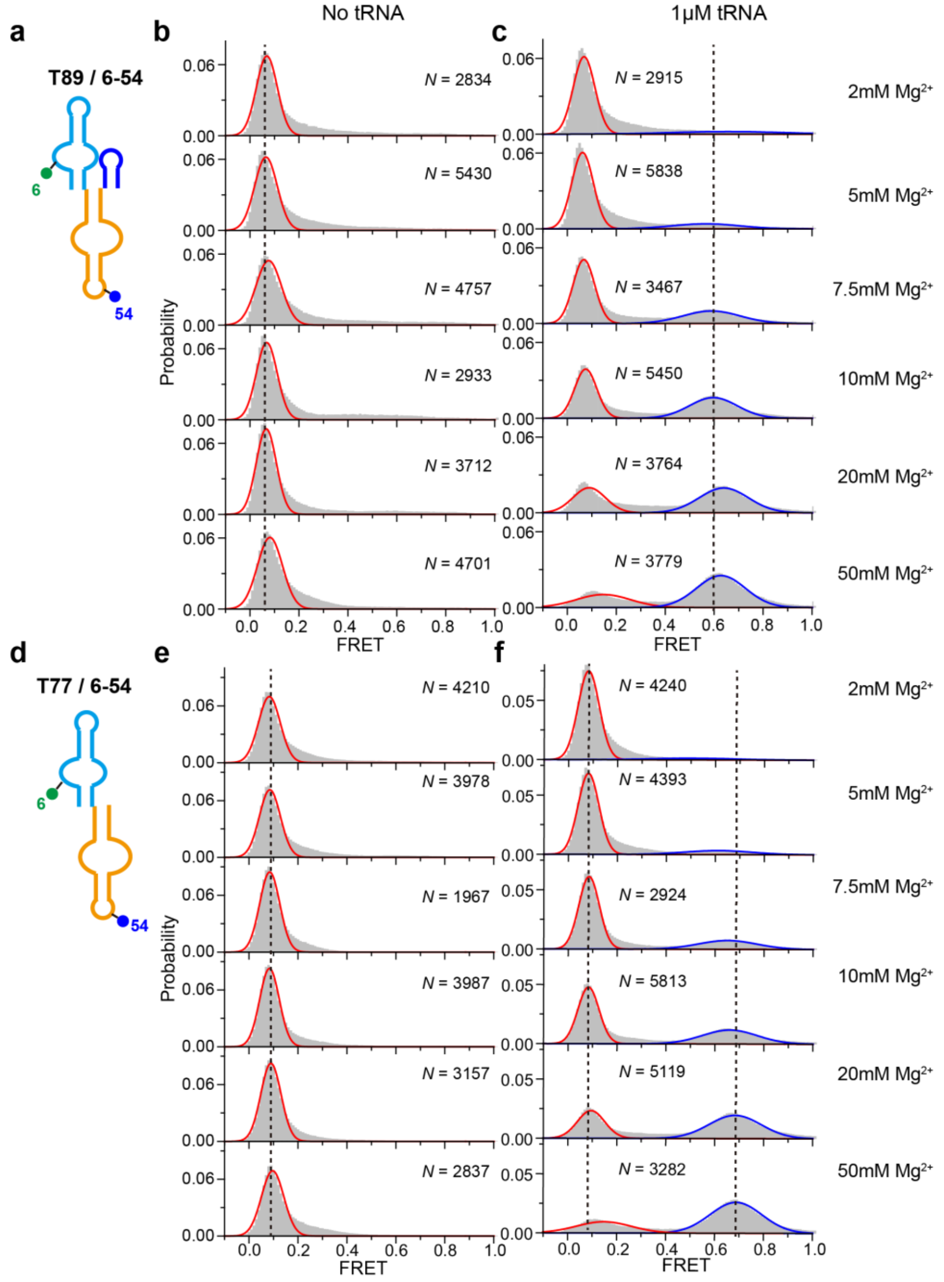

Supplementary Figure 7. **Mg^{2+}- and tRNA-dependent conformational dynamics of T89 and T77 probed by smFRET.** (**a**) Schematic representation for the secondary structure of T89/6-54 used in smFRET assay. The blue and green circle indicates Cy5 and Cy3 fluorophore, respectively. (**b-c**) FRET histograms for T89/6-54 in the absence (**b**) and presence (**c**) of tRNA across various Mg^{2+} concentrations ranging from 2 mM to 50 mM, which are related to **figure 4g-i**. (**d**) Schematic representation for the secondary structure of T77/6-54 used in smFRET assay. The red and green circles indicate Cy5 and Cy3 fluorophore, respectively. (**e-f**) FRET histograms for T77/6-54 in the absence (**b**) and presence (**c**) of tRNA across various Mg^{2+} concentrations ranging from 2 mM to 50 mM, which are related to **figure 4j-l**. Histograms were well fitted with Gaussian peaks, shown in red and blue for the low- and intermediate- FRET states, respectively. *N* denotes the total number of traces to generate histograms from three independent experiments (n = 3). Source data are provided as a Source Data file.

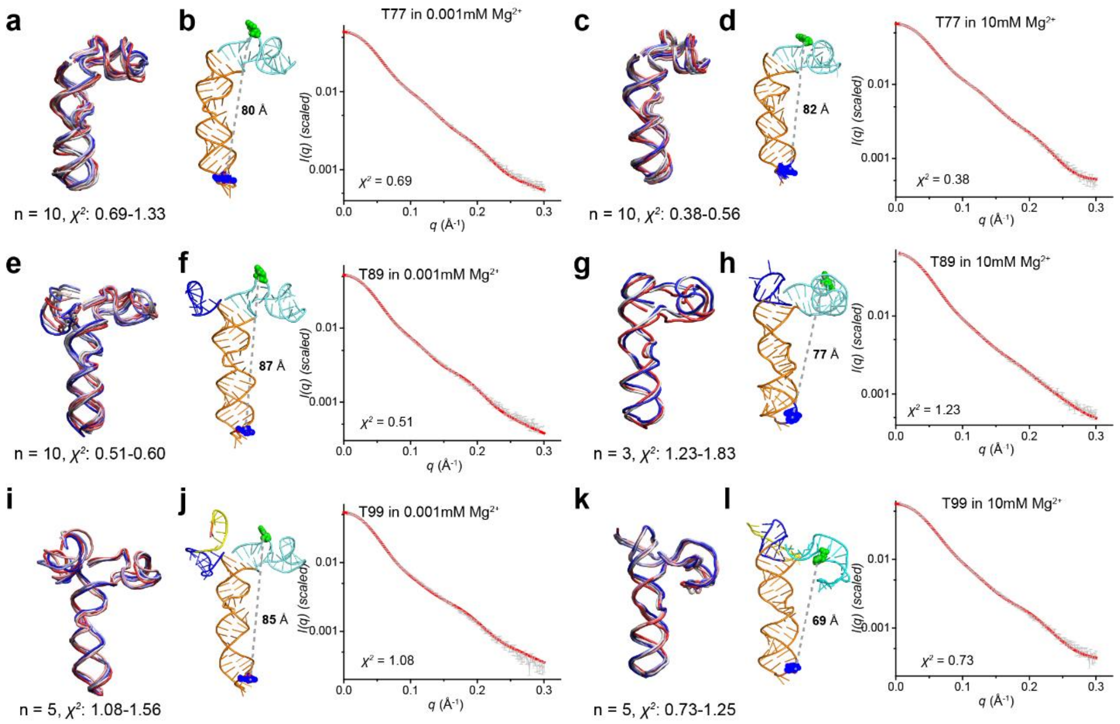

Supplementary Figure 8. The atomic 3D structural models for transcription intermediates of T99. (a, c, e, g, i, k) Superimposition of the best-fitting structural models for T77 in 0.001 mM Mg^{2+} (**a**), T77 in 10 mM Mg^{2+} (**c**), T89 in 0.001 mM Mg^{2+} (**e**), T89 in 10 mM Mg^{2+} (**g**), T99 in 0.001 mM Mg^{2+} (**i**), T99 in 10 mM Mg^{2+} (**k**). They are colored by blue-white-red spectrum according to their ranks in fitness, the more bule, the more better fitness. The number of top structural candidates and their χ^2 range are indicated. **(b, d, f, h, j, l)** Overlay of the theoretical scattering curves (red) calculated from the best-fit individual structural model (top1) with the experimental SAXS scattering curves (gray) for T77 in 0.001 mM Mg^{2+} (**b**), T77 in 10 mM Mg^{2+} (**d**), T89 in 0.001 mM Mg^{2+} (**f**), T89 in 10 mM Mg^{2+} (**h**), T99 in 0.001 mM Mg^{2+} (**j**), T99 in 10 mM Mg^{2+} (**l**). The positions of the fluorophores in the best-fit structural model (top1) were highlighted as spheres and the distances between fluorophores were indicated. All of the above structures were provided as pdb files in **Supplementary Data 2.** Source data are provided as a Source Data file.

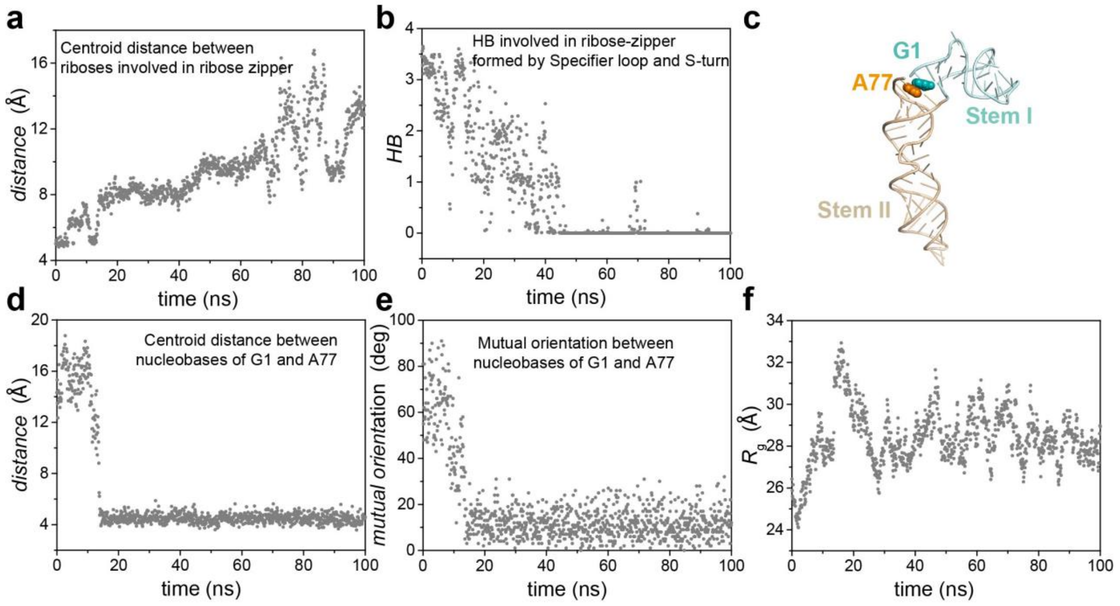

Supplementary Figure 9. Two remarkable conformational change events observed in our unbiased all-atom MD simulations. (**a-b**) Spontaneous dissociation of Specifier loop within Stem I from S-turn of Stem II in the simulation of T99, leading to a breakage of ribose-zipper interactions which are characterized by their centroid distance of ribose (**a**) as well as the number of hydrogen bonds (**b**). (**c-f**) Spontaneous stacking of Stem I on Stem II in simulation of T77, generating extended conformation (**c**) which is monitored by centroid distance (**d**) and mutual orientation angle (**e**) between nucleobase of G1 (Stem 1) and A77 (Stem II), and R_g of T77 (**f**). The native hydrogen bonds (HB) are calculated by a continuous function with their distance.The centroid distance is defined by the distance between centroid of two groups, whereas the mutual orientation angle of nucleobase is defined by the angle formed by the normal of nucleobase planes. Source data are provided as a Source Data file.

Supplementary Table 1. ITC parameters for $tRNA^{Ile}$ binding to the aptamer of *ileS* T-box riboswitch in different Mg^{2+} concentrations.

Mg^{2+} (mM)	K_d (μM)	n	ΔH (kcal/mol)	-ΔTS (kcal/mol)
0.001	n.d.	n.d.	n.d.	n.d.
1	n.d.	n.d.	n.d.	n.d.
2	0.799	0.41	-49.1	40.8
5	0.270	0.87	-25.8	16.9
10	0.080	0.88	-23.5	13.9

Supplementary Table 2. The primary sequences of RNAs used in this study.

Construct	RNA sequences
T99	5'-GGCGACGAUCCGGCCAUCACCGGGGAGCCUUCGGAAGAAC GGCGCCACCGCCCGCGGCGGCGCUCAGUAGAACCGAACGGGUGA GCCCGUCACAGCUCG-3'
T89	5'-GGCGACGAUCCGGCCAUCACCGGGGAGCCUUCGGAAGAAC GGCGCCACCGCCCGCGGCGGCGCUCAGUAGAACCGAACGGGUGA GCCCG-3'
T77	5'-GGCGACGAUCCGGCCAUCACCGGGGAGCCUUCGGAAGAAC GGCGCCACCGCCCGCGGCGGCGCUCAGUAGAACCGAA-3'
T99-A69U	5'-GGCGACGAUCCGGCCAUCACCGGGGAGCCUUCGGAAGAAC GGCGCCACCGCCCGCGGCGGCGCUCAGU**U**GAACCGAACGGGUGA GCCCGUCACAGCUCG-3'
T99-G85C	5'-GGCGACGAUCCGGCCAUCACCGGGGAGCCUUCGGAAGAAC GGCGCCACCGCCCGCGGCGGCGCUCAGUAGAACCGAACGGGUGA **C**CCCGUCACAGCUCG-3'
T99-U90C	5'-GGCGACGAUCCGGCCAUCACCGGGGAGCCUUCGGAAGAAC GGCGCCACCGCCCGCGGCGGCGCUCAGUAGAACCGAACGGGUGA GCCCG**C**CACAGCUCG-3'
MtbT87	5'-GGCGACGAUCCGGCGAUCACCGGGGAGCCUUCGGAAGAAC GGCCGGUUAGGCCCAGUAGAACCGAACGGGUUGGCCCGUCACAG CCU-3'
tRNA	5'-GGGCCUAUAGCUCAGGCGGUUAGAGCGCUUCGCUGAUAAC GAAGAGGUCGGAGGUUCGAGUCCUCCUAGGCCCGCCA-3'

(Note: the mutated residues are highlighted in bold and red.)

Supplementary Table 3. The natural and unnatural oligonucleotide primers used in this study.

Primer	Sequences
T99-WT-F	5'-CACATTTCCCCGAAAAGTGCCACCTGACGT-3'
T99-WT-R	5'-CGAGCTGTGACGGGCTCACCCGTTCG-3'
T89-WT-R	5'-CGGGCTCACCCGTTCGGTTCTACTGAGC-3'
T77-WT-R	5'-TTCGGTTCTACTGAGCGCCGCC-3'
T99-A69U-R	5'-CGAGCTGTGACGGGCTCACCCGTTCGGTTCAACTGAG-3'
T99-G85C-R	5'-CGAGCTGTGACGGGGTCACCCGTTCGGTTCTACTGAGC-3'
T99-U90C-R	5'-CGAGCTGTGGCGGGCTCACCCGTTCGGTTC-3'
T99-WT+linker-R	5'-CGCACCGGACCTCTCGTTGCGAGCTGTGA-3'
T99-C6NaM-R	5'-TCTTCCGAAGGCTCCCCGGTGATGGCCGGATC**(NaM)**TC-3'
T99-G54NaM-F	5'-ACCGGGGAGCCTTCGGAAGAACGGCGCCACCGCCC**(NaM)** C-3'
T99-C14NaM-R	5'-GTTCTTCCGAAGGCTCCCCGGTGATG**(NaM)**CCGGATCGTC GCCTATAGTG-3'
T99-S2-F	5'-CGGGGAGCCTTCGGAAGAACGGCGCCACCG-3'
T99-G99TPT3+linker-R	5'-CGCACCGGACCTCTCGTTG**(TPT3)**GAGCTG-3'
T99-G99TPT3 /T82NaM+linker	5'-CGCACCGGACCTCTCGTTG**(TPT3)**GAGCTGTGACGGGCTC **(NaM)**CCCGTT-3'
T99-G85C-G99TPT3 /T82NaM+linker	5'-CGCACCGGACCTCTCGTTG**(TPT3)**GAGCTGTGACGGGG TC**(NaM)**CCCGTT-3'
MtbT87-WT-F	5'- GTAAAACGACGGCCAGTCCGTCTCTA-3'
MtbT87-T71TPT3-R	5'-TGAGGCTGTGACGGGCCA**(TPT3)**CCCGTTCGGTTCTACT-3'
MtbT87+linker-R	5'-CTGCGTGCCTGCAATCCAGTGAGGCTGTGACGGGCC-3'
DNA-linker (T99)	5'biotin-CGCACCGGACCTCTCGT-3'
DNA-linker (T87)	5'biotin- CTGCGTGCCTGCAATCCAG-3'Cy5

Supplementary Table 4. Basic structural parameters derived from SAXS data for the wild type and mutants of T-box riboswitch.

RNAs	Mg^{2+} (mM)	$^{a}R_g$ (Å)	$^{b}D_{max}$ (Å)	^{c}MW (KDa)	^{d}MW (KDa)
T99	0.001	32.3±0.2	118	32.24	35.88
	0.01	32.4±0.2	117		35.43
	0.1	32.1±0.1	115		32.48
	0.2	31.9±0.2	115		33.18
	0.5	30.4±0.1	102		34.52
	1	30.2±0.1	102		33.74
	5	28.5±0.1	95		34.13
	10	29.4±0.1	98		34.10
T89	0.001	31.4±0.2	112	29.06	31.84
	0.01	31.6±0.2	110		32.80
	0.1	31.4±0.2	113		31.78
	0.2	31.6±0.3	113		31.11
	0.5	30.4±0.2	102		31.12
	1	30.8±0.2	103		31.49
	5	28.4±0.1	95		32.03
	10	29.4±0.1	100		31.02
T77	0.001	29.7±0.2	99	25.13	26.97
	0.01	29.2±0.2	99		28.14
	0.1	28.8±0.1	96		27.45
	0.2	29.1±0.2	98		27.26
	0.5	29.0±0.2	96		27.32
	1	28.8±0.2	95		26.56
	5	28.8±0.2	96		26.08
	10	28.5±0.2	94		26.56
T99-A69U	5	29.4±0.2	105	32.24	35.60
T99-G70U	5	28.6±0.2	95		31.70
T99-U90C	5	32.0±0.2	125		35.84
T99-G85C	5	34.1±0.3	123		38.62
T99/tRNA complex	10	34.3±0.1	125	54.14	57.28

[a]derived from Guinier fitting;
[b]derived from GNOM analysis;
[c]MW: molecular weight predicted from sequences;
[d]MW: molecular weight calculated based on the power law of volume of correlation;
All the experiments were conducted in the buffer containing 20mM Tris (pH7.5), 100mM KCl and supplemented with different concentrations of Mg^{2+}.

Supplementary Table 5. smFRET efficiencies, populations and transition rates for T99 WT in the presence of tRNA.

RNA	Mg^{2+} (mM)	FRET efficiency[a]		FRET state population[b] (%)		Transition rate[c] (s^{-1})	
		L[d]	I[e]	L[d]	I[e]	k_{L-I}	k_{I-L}
WT/6-54	1	0.16	0.59	90.0	10.0	0.16±0.03	0.60±0.04
	2	0.18	0.56	82.1	17.9	0.150±0.006	0.38±0.03
	3	0.22	0.59	59.9	40.1	0.31±0.02	0.24±0.02
	4	0.24	0.59	46.9	53.1	0.58±0.08	0.19±0.02
	5	0.25	0.58	41.9	58.1	0.70±0.09	0.21±0.01
	7.5	0.28	0.60	32.7	68.3	1.8±0.2	0.23±0.02
	10	0.28	0.58	36.3	63.7	1.9±0.5	0.20±0.01
	20	0.34	0.60	32.2	67.8	2.4±0.4	0.25±0.03

All the experiments were conducted in the buffers containing 50 mM HEPES (pH 7.5), 100 mM KCl and different concentrations of Mg^{2+}.

All results were averages of three independent experiments (n = 3) and presented as mean ± SEM. [a] SEM (standard error of the mean) is 0.01 or lower; [b] SEM is 1% or lower; [c] SEM is listed. [d] Low FRET; [e] Intermediate FRET.

Supplementary Table 6. smFRET efficiencies, populations and transition rates of T99 mutants in the presence of 1μM tRNA.

RNA	Mg^{2+} (mM)	FRET efficiency[a]		FRET state population (%)[b]		Transition rate (s^{-1})[c]	
		L[d]	I[e]	L[d]	I[e]	k_{L-I}	k_{I-L}
T77/6-54	2	0.08	0.50	93.1	6.9	0.093±0.009	-
	5	0.08	0.61	86.1	13.9	0.113±0.009	0.51±0.03
	7.5	0.09	0.66	71.9	28.1	0.21±0.02	0.36±0.02
	10	0.08	0.66	60.8	39.2	0.263±0.003	0.30±0.01
	20	0.10	0.68	38.6	61.4	0.51±0.01	0.211±0.002
	50	0.15	0.69	28.9	71.1	1.08±0.03	0.130±0.008
T89-6/54	2	0.07	-	85.4	14.6	-	-
	5	0.06	0.57	83.2	16.8	0.113±0.007	0.42±0.04
	7.5	0.07	0.59	65.1	34．9	0.27±0.01	0.23±0.01
	10	0.08	0.60	52.6	47.4	0.240±0.003	0.23±0.01
	20	0.09	0.64	40.4	59.6	0.491±0.004	0.17±0.04
	50	0.15	0.63	31.2	68.8	1.01±0.07	0.14±0.03
G85C/6-54	7.5	0.25	0.68	48.2	51.8	1.1±0.1	0.31±0.04
U90C/6-54	7.5	0.28	0.67	53.2	46.8	1.07±0.04	0.313±0.003
G70U-6/54	20	0.27	0.67	27.0	73.0	0.51±0.02	0.59±0.01

All the experiments were conducted in the buffers containing 50 mM HEPES (pH 7.5), 100 mM KCl and different concentrations of Mg^{2+}.

All results were averages of three independent experiments (n = 3) and presented as mean ± SEM. [a] SEM (standard error of the mean) is 0.01 or lower; [b] SEM is 1% or lower; [c] SEM is listed. [d] Low FRET; [e] Intermediate FRET.

Supplementary Table 7. The list of RNA constructs with different configuration for MD simulation.

construct	configuration index for each construct	Description of initial configuration for MD simulation	Total number of atoms
T99	1	Just removing tRNA from 6UFM	177803
	2	Stem I is undocked	177515
	3	Stem I is undocked, stem IIA is stacked on stem II and the pseudoknot of stem IIA/B is not formed.	187813
	4	Stem I is stacked on stem II and the pseudoknot of stem IIA/B is not formed	198701
T89	1	Stem I is undocked and stem IIA is stacked on stem II	155059
	2	Stem I is stacked on stem II	164623
T77	1	Stem I is undocked	153824

Note: All of the above structures used to initiate MD simulations were provided as pdb files in **Supplementary Data 1** and named as T99.1-T99.4, T89.1-T89.2, T77.1.

Supplementary References

1. Wang, X., Unarta, I.C., Cheung, P.P. & Huang, X. Elucidating molecular mechanisms of functional conformational changes of proteins via markov state models. *Curr Opin Struct Biol* **67**, 69-77 (2021).
2. Watkins, A.M., Rangan, R. & Das, R. FARFAR2: Improved de novo Rosetta prediction of complex global RNA folds. *Structure* **28**, 963 (2020).
3. Cornell, W.D. et al. A second generation force field for the simulation of proteins, nucleic acids, and organic molecules. *J. Am. Chem. Soc* **118**, 2309-2309 (1996).
4. Cheatham, T.E., 3rd, Cieplak, P. & Kollman, P.A. A modified version of the Cornell et al. force field with improved sugar pucker phases and helical repeat. *J Biomol Struct Dyn* **16**, 845-62 (1999).
5. Perez, A. et al. Refinenement of the AMBER force field for nucleic acids: Improving the description of alpha/gamma conformers. *Biophys J* **92**, 3817-3829 (2007).
6. Zgarbova, M. et al. Refinement of the Cornell et al. Nucleic acids force field based on reference quantum chemical calculations of glycosidic torsion profiles. *J Chem Theory Comput* **7**, 2886-2902 (2011).
7. Steinbrecher, T., Latzer, J. & Case, D.A. Revised AMBER parameters for bioorganic phosphates. *J Chem Theory Comput* **8**, 4405-4412 (2012).
8. Izadi, S., Anandakrishnan, R. & Onufriev, A.V. Building water models: A different approach. *J Phys Chem Lett* **5**, 3863-3871 (2014).
9. Li, Z., Song, L.F., Li, P. & Merz, K.M., Jr. Systematic parametrization of divalent metal Ions for the OPC3, OPC, TIP3P-FB, and TIP4P-FB water models. *J Chem Theory Comput* **16**, 4429-4442 (2020).
10. Sengupta, A., Li, Z., Song, L.F., Li, P. & Merz, K.M., Jr. Parameterization of monovalent ions for the OPC3, OPC, TIP3P-FB, and TIP4P-FB water models. *J Chem Inf Model* **61**, 869-880 (2021).
11. Shabane, P.S., Izadi, S. & Onufriev, A.V. General purpose water model can improve atomistic simulations of intrinsically disordered proteins. *J Chem Theory Comput* **15**, 2620-2634 (2019).
12. Abraham, M.J. et al. GROMACS: High performance molecular simulations through multi-level parallelism from laptops to supercomputers. *SoftwareX* **1-2**, 19-25 (2015).
13. Parrinello, M. & Rahman, A. Polymorphic transitions in single crystals: A new molecular dynamics method. *J. Appl. Phys* **52**, 7182-7190 (1981).
14. Bussi, G., Donadio, D. & Parrinello, M. Canonical sampling through velocity rescaling. *J. Chem. Phys* **126**(2007).
15. Hess, B., Bekker, H., Berendsen, H.J.C. & Fraaije, J.G.E.M. LINCS: A linear constraint solver for molecular simulations. *J Comput Chem* **18**, 1463-1472 (1997).
16. Darden, T., York, D. & Pedersen, L. Particle mesh Ewald - An *N*.Log(*N*) method for Ewald sums in large systems. *J. Chem. Phys* **98**, 10089-10092 (1993).
17. Svergun, D., Barberato, C. & Koch, M.H.J. CRYSOL - A program to evaluate x-ray solution scattering of biological macromolecules from atomic coordinates. *J Appl Crystallogr* **28**, 768-773 (1995).
18. Noel, J.K. et al. SMOG 2: A versatile software package for generating structure-based models. *PLoS Comput Biol* **12**, e1004794 (2016).
19. Whitford, P.C. et al. An all-atom structure-based potential for proteins: bridging minimal models with all-atom empirical forcefields. *Proteins* **75**, 430-41 (2009).
20. Niu, X. et al. Molecular mechanisms underlying the extreme mechanical anisotropy of the flaviviral exoribonuclease-resistant RNAs (xrRNAs). *Nat Commun* **11**, 5496 (2020).
21. Humphrey, W., Dalke, A. & Schulten, K. VMD: Visual molecular dynamics. *J Mol Graph Model* **14**, 33-38 (1996).
