# Supplementary material for: Structural and dynamic mechanisms for coupled folding and tRNA recognition of a translational T-box riboswitch
Source: Nat Commun. 2023 Nov 15;14:7394. doi: 10.1038/s41467-023-43232-z (PMC10651847; doi:10.1038/s41467-023-43232-z)
Supplement: Supplementary file 3 — Description of Additional Supplementary Files [file 41467_2023_43232_MOESM3_ESM.pdf]

Description of Additional Supplementary Files

File name: Supplementary Data 1
Description: Initial structural models for MD simulation.

File name: Supplementary Data 2
Description: Atomic structural models for T77, T89 and T99 in different Mg^{2+} concentrations.

File name: Supplementary Movie 1
Description: Mg^{2+}-induced conformational change of T99. Stem I, stem II, stem IIA and 3' tail of T99 was shown as Cyan, orange, blue and yellow ribbon, respectively. In low Mg^{2+} (at the beginning), stem IIA/B pseudoknot is unfolded and stem I coaxially stacks with stem II, T99 mainly samples "undocked" state; Mg^{2+} promote the stabilization and folding of stem IIA/B pseudoknot, then the stem IIA/B tends to coaxially stacks with stem II and promote the directional motion of stem I toward stem II, making stem I being close to stem II but doesn't form any tertiary interactions, we defined this state as "pre-docked" state (at the ending).

File name: Supplementary Movie 2
Description: tRNA-induced conformational change of T99. Stem I, stem II, stem IIA/B of T99 were shown as Cyan, orange and blue ribbon, respectively. tRNA was shown as green ribbon. The Specifier loop in stem I, S-turn region in stem II and tRNA anticodon were highlighted as magenta. In high Mg^{2+} and absence of tRNA (at the beginning), T99 mainly samples the "pre-docked" state, in which the stem I is close to stem II but the Specifier-S-turn binding cleft is not formed; In the presence of tRNA, tRNA anticodon presumably contact with the Specifier loop in stem I firstly. This will drive the formation of a helical, stacked conformation for the specifier. Then stem I docks with stem II via backbone interactions with the S-turn region, which in turn reinforces the Specifier-anticodon interactions. Thus, T99 mainly samples the "docked" state *in holo* form (at the ending).
